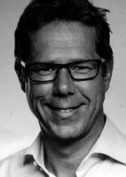# Room‐Temperature Laser Synthesis in Liquid of Oxide, Metal‐Oxide Core‐Shells, and Doped Oxide Nanoparticles

**DOI:** 10.1002/chem.202000686

**Published:** 2020-07-01

**Authors:** Vincenzo Amendola, David Amans, Yoshie Ishikawa, Naoto Koshizaki, Salvatore Scirè, Giuseppe Compagnini, Sven Reichenberger, Stephan Barcikowski

**Affiliations:** ^1^ Department of Chemical Sciences University of Padova Via Marzolo 1 35131 Parova Italy; ^2^ CNRS Institut Lumière Matière Univ Lyon, Université Claude Bernard Lyon 1; ^3^ Nanomaterials Research Institute National Institute of Advanced Industrial Science and Technology (AIST) Tsukuba Central 5, 1-1-1 Higashi Tsukuba Ibaraki 305-8565 Japan; ^4^ Graduate School of Engineering Hokkaido University Kita 13 Nishi 8, Kita-ku Sapporo Hokkaido 060-8628 Japan; ^5^ Department of Chemical Sciences University of Catania Viale A. Doria 6 Catania 95125 Italy; ^6^ Technical Chemistry I and Center for Nanointegration Duisburg-Essen (CENIDE) University Duisburg-Essen Universitätstr. 7 45141 Essen Germany

**Keywords:** core–shell nanoparticles, doped oxides, laser ablation in liquid, laser fragmentation in liquid, laser melting in liquid, oxide nanoparticles

## Abstract

Although oxide nanoparticles are ubiquitous in science and technology, a multitude of compositions, phases, structures, and doping levels exist, each one requiring a variety of conditions for their synthesis and modification. Besides, experimental procedures are frequently dominated by high temperatures or pressures and by chemical contaminants or waste. In recent years, laser synthesis of colloids emerged as a versatile approach to access a library of clean oxide nanoparticles relying on only four main strategies running at room temperature and ambient pressure: laser ablation in liquid, laser fragmentation in liquid, laser melting in liquid and laser defect‐engineering in liquid. Here, established laser‐based methodologies are reviewed through the presentation of a panorama of oxide nanoparticles which include pure oxidic phases, as well as unconventional structures like defective or doped oxides, non‐equilibrium compounds, metal‐oxide core–shells and other anisotropic morphologies. So far, these materials showed several useful properties that are discussed with special emphasis on catalytic, biomedical and optical application. Yet, given the endless number of mixed compounds accessible by the laser‐assisted methodologies, there is still a lot of room to expand the library of nano‐crystals and to refine the control over products as well as to improve the understanding of the whole process of nanoparticle formation. To that end, this review aims to identify the perspectives and unique opportunities of laser‐based synthesis and processing of colloids for future studies of oxide nanomaterial‐oriented sciences.

## Graphical abstract

**Laser‐assisted synthesis** of colloids emerged as a versatile approach to access a library of oxide nanoparticles and related nanostructures at room temperature and ambient pressure. This review provides a critical presentation of laser‐based methodologies, of the various oxide nanoparticles achieved so far, and of their applications in catalysis, optics, biotechnology and further emerging fields.

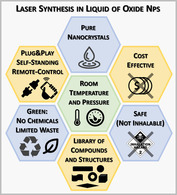

## Introduction

Oxide nanoparticles (NPs) are largely exploited for a variety of purposes, which embraces fields as different as, for instance, heterogeneous catalysis, biotechnology, medicine, photonics, solar energy conversion, microelectronics, automotive industry, pharmaceutics, and food additives.^1^, ^2^ This variety of applications also comes with a vast number of distinct compounds belonging to the class of oxide nanomaterials. The synthesis of oxides with tailored properties requires a multitude of different synthetic procedures, including for instance the hydrothermal, calcination, mini‐emulsion, spray pyrolysis, plasma‐assisted and inert‐atmosphere growth methods.^1^, ^2^, ^3^ Usually, these procedures allow high productivity but require sophisticated setups (autoclave reactors for the hydrothermal methods, furnaces for calcination, vacuum systems for plasma‐assisted and inert atmosphere methods, pressure‐ and precursor‐flow‐controlled flame synthesis). Also tailored experimental conditions including high temperature and pressure (hydrothermal, calcination, spray pyrolysis) and chemical precursors and/or additives that potentially persist as contaminants in the final products (calcination, mini‐emulsions, plasma‐assisted chemical vapor deposition) are needed. The required precursors and their synthesis, as well as the post‐treatment, often leads to toxic or pollutant waste, which poses the problem of their disposal.^3^ Moreover, for some oxide cations, no precursors are available at all, limiting their flame spray synthesis. In the framework of the global efforts towards a circular and sustainable economy, it is therefore of utmost importance to develop synthesis routes running at room temperature and ambient pressure, which allows the cost‐effective and green development of nanotechnologies based on oxides.

**Figure 1:**
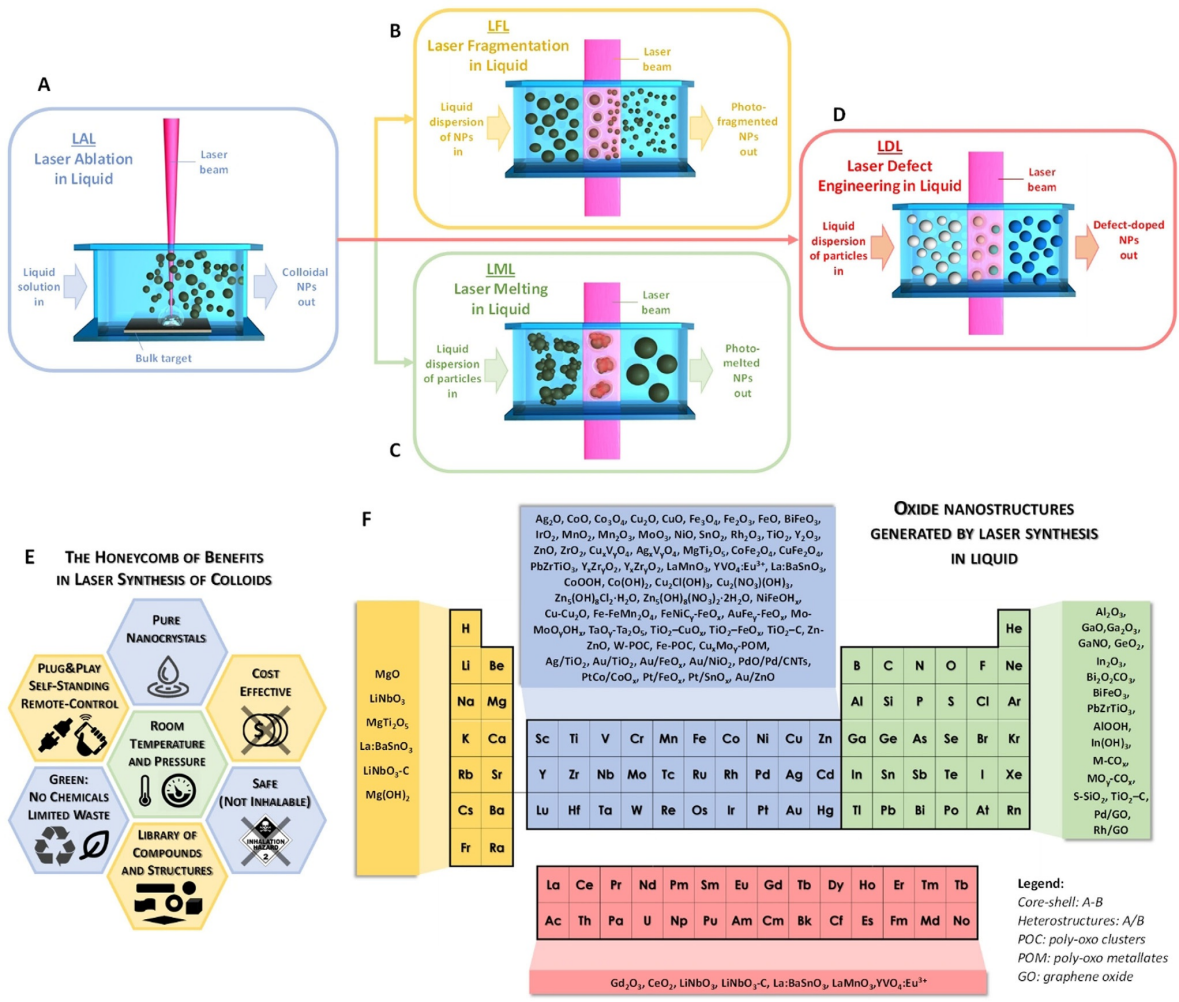
The landscape of laser synthesis of oxide nanoparticle colloids: Sketch of LAL (A), LFL (B), LML (C) and LDL (D). E) Honeycomb with frequently encountered advantages in laser synthesis of colloids. F) Overview of oxide nanostructures generated by laser synthesis in liquid in the literature.

To this end, the laser‐assisted synthesis of colloidal NPs, that is, the use of laser beams to generate a dispersion of NPs in a liquid environment, is emerging as a promising approach.^4^, ^5^, ^6^, ^7^ The oldest examples are dated back around 1991–1993,^8^, ^9^ and are based on laser ablation in liquid (LAL),^10^ where a laser beam is directed on a solid target immersed in a liquid solution, to generate a colloid through the ablation of the surface of the solid (Figure 1 A). In most cases, LAL is performed with pulsed lasers, and it is also called pulsed‐LAL (PLAL). The synthesis of nanomaterials by LAL is sometimes called laser ablation synthesis in solution (LASiS).^11^ In 1997,^12^ a variant of LAL appeared, where the laser beam is focused inside a liquid dispersion of micrometric or nanometric powders, to obtain their photo‐fragmentation into smaller NPs, in a process known as laser fragmentation in liquid (LFL, Figure 1 B).^10^, ^13^, ^14^, ^243^

Soon, LFL turned out to be also an effective way for the reduction of size and polydispersity of the colloids obtained by LAL. While performing LFL with laser pulse energy lower than the fragmentation threshold, it was observed that it is possible to obtain the photothermal fusion of aggregates of NPs into larger nanospheres,^11^, ^15^ or the photothermal melting and vaporization of micrometric powders into submicron spheres,^16^ through a process known as laser melting in liquid (LML) or pulsed‐LML (PLML), Figure 1 C.^10^, ^16^ LML was also applied to the size increase of the colloids obtained by LAL. In fact, LAL, LFL, and LML can be combined for the production and the subsequent size control (reduction or increase) of colloidal NPs, as shown already in 2007.^17^ Using milder fluence regimes to irradiate a colloid with the intention mainly to change the atomic structure of NPs by introducing defects, while keeping the size unchanged, is called laser defect‐engineering in liquid (LDL, Figure 1 D). Inspired by the first seminal reports in the field, a growing international community of scientists pursued the study of physical and chemical processes involved in laser synthesis of colloids.^6^, ^10^ They demonstrated several advantages of the method (Figure 1 E) and that colloids can be generated with peculiar features (e.g., metastable phases and doped nano‐crystals) not present in NPs obtained by other procedures, as will be described in the following text.^6^, ^10^, ^18^

1) First of all, laser‐generated colloids are highly pure, ligands‐free and expose an uncoated surface, because no chemical precursors, chelating agents or coordinating molecules are required in the majority of cases.^11^, ^19^, ^20^ Often, the absence of pollutant waste and the use of raw materials make laser synthesis compatible with the 12 principles of green chemistry,^11^ offering new opportunities for the development of a green and sustainable nanotechnology, and for the integration of colloidal NPs in a circular economy.

2) The achievement of oxide NPs as a colloid does not expose the operator to airborne particle inhalation risks, thus allowing occupational safe and easy manipulation of the products, compared to dry nano‐powders.^21^ Besides, the effective interaction of NPs in a stable colloid with solid substrates and matrixes is facilitated by impregnation or mixing with the liquid phase.^10^, ^19^

3*)* Another relevant advantage of laser‐assisted synthesis methods is the access to a wide range of oxide (and non‐oxide) nanoparticles in similar experimental conditions, all at room temperature and pressure,^18^ as shown in Figure 1 F on the basis of a literature overview. To clarify why laser synthesis falls under the *“*room‐temperature and pressure synthesis*”* classification, on the one hand, the mechanistically relevant local (temporal and spatial) temperature and pressure profiles and on the other hand, the practically relevant global‐extrinsic parameters have to be differentiated. It is worth specifying that locally and temporally, at the level of the matter interacting with the laser beam, extremely high temperatures and pressures are reached, but this is self‐confined in a limited region of space coincident with the laser spot and the early explosive boiling volume. Molecular dynamic simulations coupled with the two‐temperature‐model were recently extended from the ultrashort‐pulsed to nanosecond‐pulsed laser ablation regime,^22^ where after short non‐equilibrium phase the majority of nanoparticles are in thermodynamic equilibrium with their local environment at the end of the simulation (a few nanoseconds).

These predictions are backed by experimental findings on binary, partly immiscible nanoparticle systems, where both a kinetic control and thermodynamic contribution of the particle formation dynamics are concluded.^23^, ^24^, ^25^ Hence, on the one hand, highly non‐equilibrium conditions are pointing at a kinetic control of the synthesis at the very early, sub‐nanosecond formation time regime.^26^ Later, the whole system quickly reaches and remains in equilibrium with ambient conditions. Globally, there is no need for strategies for heat or pressure regulation, which is a big advantage compared to gas‐phase (pressure), hydrothermal (pressure, temperature), or sol‐gel (temperature) synthesis, and wet chemical co‐precipitation or reduction (temperature). Even with high repetition rate lasers (>kHz), liquid flux simultaneously works for draining the colloid and cooling the synthesis environment macroscopically keeping a steady state of temperature and pressure.

This means that laser synthesis does not require unit operations for in‐process‐heating/cooling or pressure control, which makes laser synthesis systems easily implemented in laboratories. Overall, laser synthesis mechanistically benefits from accessibility to metastable nanoparticle crystal structures or compositions via temporal, pulsed, non‐equilibrium condition that is confined in a microscopic volume, at the same time macroscopically continuously operating in steady state, at room temperature and pressure.

**Figure 2:**
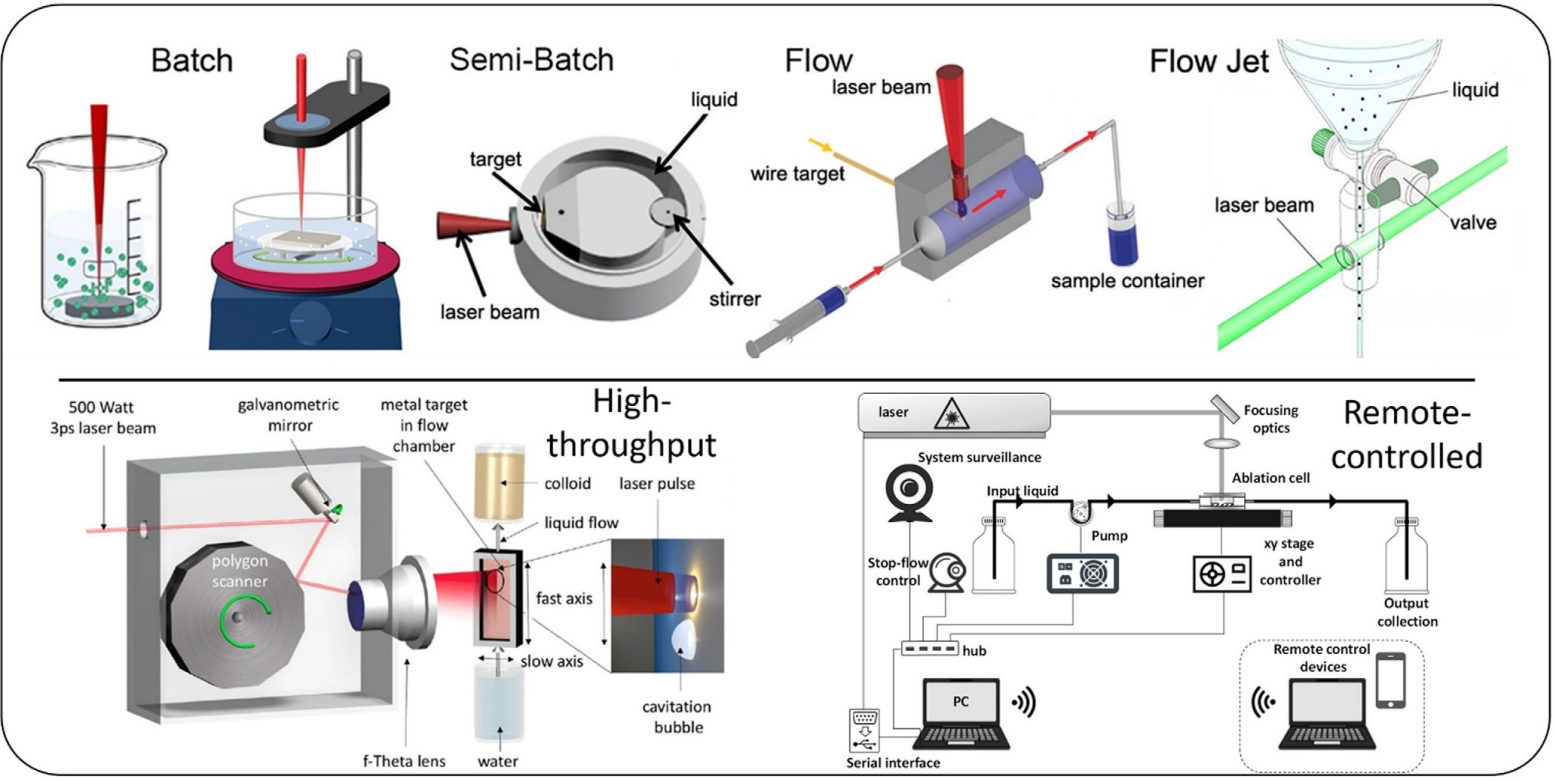
Sketch of basic and advanced set‐ups for the generation of colloids by LAL, LFL, or LML. Upper panel (from left to right, reprinted with permission from ref. 10, Copyright 2017, American Chemical Society): batch (ablation in beaker without stirring; reprinted with permission from ref. 28, Copyright 2015, American Chemical Society and ablation in beaker with stirring; reprinted with permission from ref. 29, Copyright 2015, Royal Society of Chemistry), semi‐batch (reprinted with permission from ref. 30, Copyright 2014, American Chemical Society), flow (reproduced with permission from ref. 31, Copyright 2013, Royal Society of Chemistry) and flow‐jet (Adapted with permission under CC BY 4.0 from ref. 32, Copyright 2016, Springer Nature Ltd.). The latter has been reported only for LFL and LML, see the following paragraphs. Lower panel: high throughput LAL set up with productivity up to >1 g h^−1^ (reprinted with permission from ref. 33, Copyright 2016, The Optical Society), remote‐controlled flow LAL set up with embedded stop‐flow optical control (reprinted with permission from ref. 34, Copyright 2019, AIP Publishing).

4) By using the same equipment, it is possible to synthesize NPs in a few minutes and to switch from one type of nanoparticle to another with a „plug‐and‐play“ approach.^21^

5) The experimental configuration can be tailored to the desired quantity of NPs, going from batch to flow cells (Figure 2).^10^ Flow cells have the advantage of limiting or avoiding the absorption of the laser beam by the just‐formed NPs and persistent microbubbles,^27^ is especially relevant at visible and UV wavelengths.^10^, ^18^

6) The rich library of oxide nanoparticles obtained by laser synthesis in liquid also includes non‐equilibrium phases and complex morphologies such as core–shell NPs, dendrites, spindles and, in specific cases, also nanowires, nanoflakes, nanoflowers, urchins, rods, sheets, and hollow spheres.^18^, ^35^ As explained in the following paragraphs, particle formation after each single laser pulse occurs on a timescale of 10^−6^ s,^26^ making possible the freezing of non‐equilibrium phases or highly defective structures otherwise difficult to achieve.^19^, ^36^, ^37^, ^38^

7) A feature making laser synthesis very appealing for research and industrial exploitation is that the method is intrinsically self‐standing and can be implemented with minimal manual operation. For instance, it was recently demonstrated, that LASiS of various NPs is possible by controlling the equipment remotely with a PC or a smartphone.^34^ This is useful for an even more economic synthesis also for safer synthesis conditions as it minimizes operator permanence in nearby of the laser beam, flammable solvents or harmful compounds such as radioactive elements or volatile organic molecules, even when dealing with non‐toxic oxide NPs.^34^

Further developments in the automation of laser synthesis have been recently reported by real‐time correlation of NP productivity with acoustic emission energy,^39^ as well as temperature in the ablation chamber.^40^

8) Laser synthesis in liquid is a linearly scalable method (productivity linearly scales with both laser power and time, at liquid flow operation), not yet demonstrated for production of kilograms of NPs or more.^10^, ^18^ Nonetheless, the use of a high‐power ultrafast laser with MHz repetition rate coupled with a polygon scanner (that achieves bypassing cavitation bubbles by supersonic lateral beam displacement) and a galvanometric mirror led to several grams/hour productivity of NPs by LAL, in a self‐standing continuous flow set‐up.^33^, ^41^

9) Laser synthesis is generally considered an economically viable approach in the case of NPs involving precious metals or expensive compounds.^42^ Its economic viability is strongly dependent on material type. As a rule of thumb, the LAL productivity scales with the material density,^43^ making the lighter oxides less productive than, for example, the noble metals. Of course, also the ablation threshold fluence (often higher for oxides), the electron‐phonon coupling as well as the (temperature‐dependent) target reflectivity contribute to laser ablation efficiency and thereby the power‐specific productivity. But more material‐specific factors contribute to the overall cost, including raw materials (bulk solids, solvents), hourly labour costs (manpower), the depreciation of the investment (equipment and its maintenance), the rental cost of the facilities, storage of products and the quality control of the whole procedure. The bottleneck for economic scale‐up can only be identified for a particular business case, but laser synthesis access to hundreds of different materials, making the detailed comparison of economic viability for each of them out of the scope of this review. The commercial interest in laser‐generated colloids is demonstrated by the existence of well‐established companies in Germany, Israel, and the U.S., commercializing this type of product for more than a decade.^44^, ^45^, ^46^ Besides, a few studies specifically addressed the case of laser‐generated colloids and afforded some of the parameters in the previous list. Benchmarking between hydrothermal process, photochemistry and laser ablation in liquids has been conducted for low‐priced oxide NPs (cerium oxide) dedicated to organophosphorus degradation.^47^ On the one hand, LAL was the most expensive method in this study on oxide nanoparticles, because of the usage of a not state‐of‐the‐art laser setup with limited productivity of only 21 mg h^−1^ and the relatively high depreciation of the investment (equipment). On the other hand, LAL‐generated CeO_2_ NPs exhibited the best degradation activity because of the minor surface contamination inherent to the LAL‐generated NPs and were the only one amenable to in situ production without the need for high‐temperature ovens. Conversely, for gold colloids, it has been calculated that the break‐even point where laser synthesis beats chemical synthesis in the costs versus the mass of produced NPs plot already happens at tens of grams.^42^ This is possible for the limited manual operation, absence of expensive chemical precursors, and continuous advancement in synthesis scale‐up. Interestingly, the lower cost of waste management was not even considered in the study. In both cases, the main source of cost in laser synthesis is connected to the laser equipment, that in the last 15 years showed a continuous growth of average power available on the market, and a parallel decay of the equipment cost per watt,^10^ suggesting a favourable prospect for further reduction of production costs in case of laser‐assisted synthesis of colloids.

In this review article, we provide the working principles behind LAL, LFL, LML, and LDL focusing on oxide nanostructures, with an overview of nanomaterials produced so far by this method, and of their functional properties and reported applications. So far key reviews have mainly focused on metals, alloys or processing variants during LSPC, while oxides were mentioned only peripherally.^5^, ^10^, ^19^, ^36^, ^48^, ^49^ By definition, an oxide nanomaterial consists of a nanoscale object including oxygen atoms in its chemical formula. Generally, in this review, oxide materials are defined as inorganic compounds obtained by reaction of oxygen with an element with low reduction potential. The redox potential changes with environmental parameters such as solvent, solute (concentration), temperature and pressure and, in fact, also noble metals can be surface‐oxidized in some laser synthesis conditions.^19^ However, this is usually limited to a minority of the atoms in the NPs, therefore the chemical composition of the resulting material only contains a relatively small amount of oxygen. Hence, laser‐generated and laser‐processed nanomaterials that only express limited oxidation are excluded by this review, which deals only with the compounds where the content of oxygen is comparable to that of the other main elements.

It is expected that the research on laser synthesis for the preparation of oxide nanoparticles in liquids will continue to grow in the near future, especially if one considers the variety in terms of composition and structures that are achievable, the interest in better control of the composition, size, morphology, and phase, and the need for improving the understanding of NP formation mechanism. To support this development, a non‐profit conference series on Advanced Nanoparticle Generation and Excitation by Lasers in liquids (ANGEL)^50^ exists since more than a decade, and handbooks about laser synthesis and processing of colloids are available through open access for beginners,^21^ in addition to multiple specific and advanced review articles that appeared in recent years.^10^, ^11^, ^16^, ^18^, ^19^, ^20^, ^26^, ^35^, ^36^, ^48^, ^52^, ^53^, ^54^

In the following, the review will introduce the fundamental concepts of LAL and give an overview and discussion of the work and perspectives of laser‐based synthesis of conventional nano‐oxides, core–shell NPs, defect‐engineered oxide NPs, and ligand‐stabilized oxide NPs. The discussion will include also the synthesis of multicomponent oxide nanostructures by sequential LAL and reactive LAL, highlighting the issues encountered with the compositional homogeneity of the products. Subsequently, the fundamentals and an overview of oxide NPs obtained by LFL and LML as well as upscaling considerations will be treated. Progress in the recently established laser‐based defect engineering in liquid (LDL) will be also discussed.

Similar to LFL and LML, the LDL method treats dispersed particles, with comparable mild excitation conditions, but not primarily intending to downsize particles (like LFL) or total particle melting (like LML). Instead, LDL aims at defect introduction, also relevant for the preparation of (surface‐)doped oxide NPs. The review is concluded by an overview of recent, most relevant applications of laser‐generated oxide nanomaterials, with special emphasis on photo‐catalysis, oxidation‐catalysis, bio‐applications, and photonics.

## LAL

### LAL fundamentals

**Figure 3:**
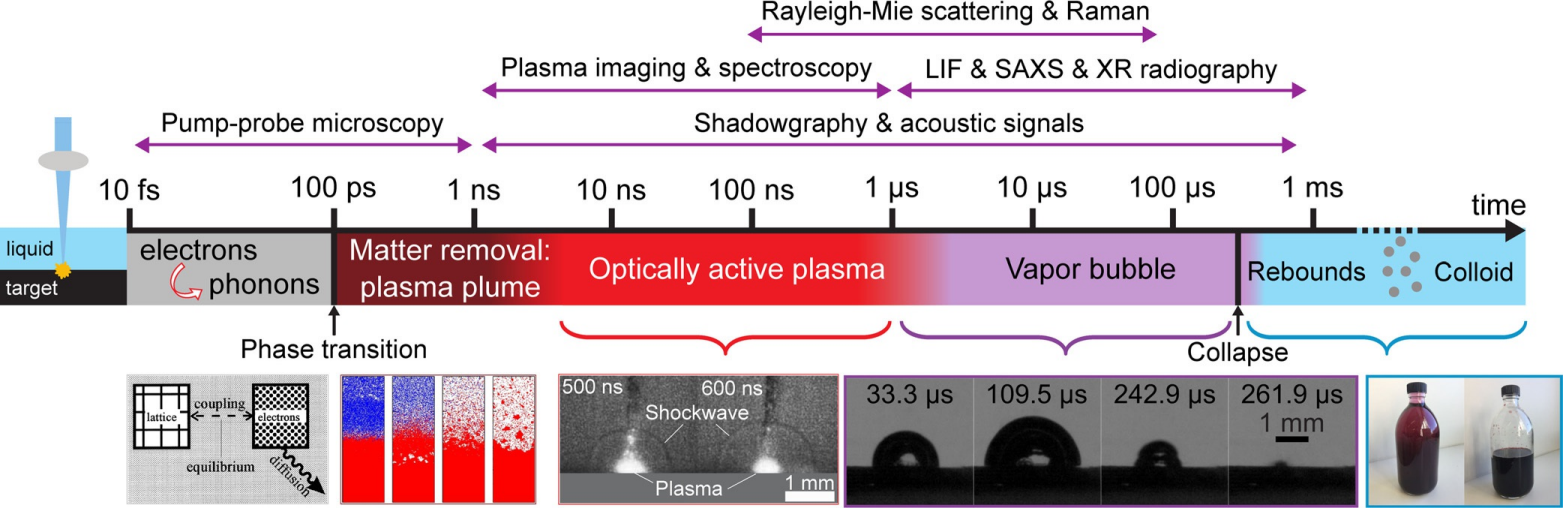
The timeline in LAL for ultrashort pulses shows the successive steps that occur during LAL synthesis, from the laser pulse interaction with the target to the release of the as‐produced NPs in the solution. On top, the characterization techniques are displayed according to their temporal resolution. At the bottom, from left to right: electron‐phonon coupling scheme, phase transition snapshot from molecular dynamics simulation, optically active plasma and released shockwave, bubble dynamics and produced colloidal solution. Reprinted with permission from ref. 60, Copyright 2017, Elsevier.

Before entering a more insightful description of the ablation mechanisms, it is useful to consider first the major steps of the most prominent laser synthesis method, the LAL (Figure 3). Starting from the general LAL configuration where a laser beam is focused on a bulk target immersed in a liquid, first of all, the laser beam should travel through a low‐absorbing liquid layer, which means that the liquid must be transparent at the chosen laser wavelength, and liquid breakdown must be avoided at the fluence selected for the experiment.^10^, ^18^ This issue is common to LAL, LFL and LML as well, and it becomes especially challenging when using ultrashort pulses, due to the self‐focusing and filamentation effects.^55^, ^56^, ^57^, ^58^, ^59^

**Figure 4:**
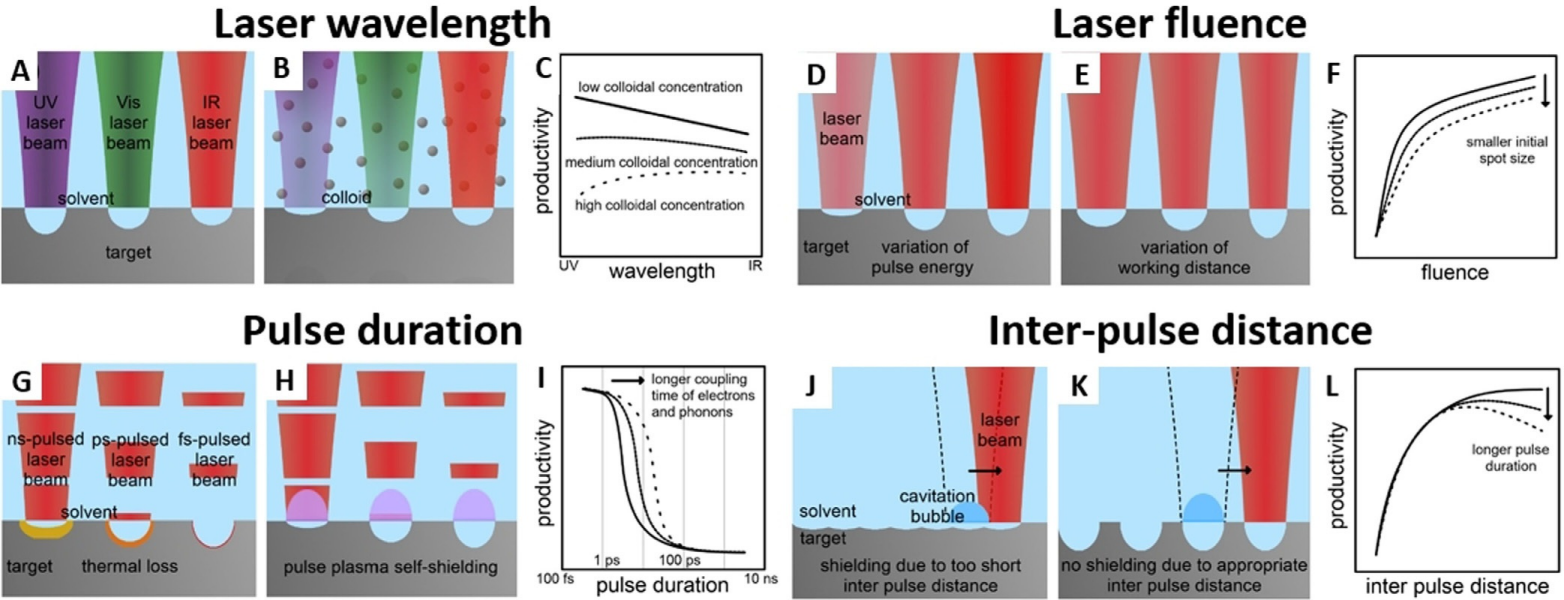
Illustrations on the change of nanoparticle productivity with the applied laser parameters; (A–C) laser wavelength; (d–F) incident laser fluence; (G–I) laser pulse duration (single‐pulse productivity) and (J–L) inter‐pulse distance. The left pictures (A, D, G, J) illustrate the effect of the respective parameter in the absence of NPs; the middle (B, E, H, K) in the presence of NPs during laser ablation. Illustrations to the right (C, F, I, L) show generalized trends occurring to productivity when varying the respective laser parameter while keeping the other parameters constant. (adapted with permission from ref. 80).

Assuming the requisite of liquid transparency, the interaction of the laser beam with the bulk target results in the formation of a plasma in a few hundreds of picoseconds.^60^, ^61^, ^62^, ^63^, ^64^, ^65^ The plasma is initially made of the target material. Because of the fast volume expansion (appearing already after about 10–100 ps),^57^, ^66^ a mechanical shockwave is released both in the target and in the liquid (Figure 3).^67^, ^68^, ^69^, ^70^, ^71^ Such a shockwave can lead to phase transition at the target and the pressure at the focal point can reach a few gigapascals.^60^ On the other hand, the interaction of the plasma with the liquid leads to the fast vaporization of the liquid, which is observed already at the shortest gating times (few ns) of CCD cameras commonly used to observe plasma dynamics. A cavitation bubble is initiated and appears mainly composed of the vaporized solvent.^61^, ^71^, ^72^, ^73^ As depicted in Figure 3, the cavitation bubble grows and collapses after a characteristic time depending on the pulse energy and the pulse duration (typically a hundred microseconds for nanosecond pulses of a few mJ). When the cavitation bubble collapses, the NPs are released in the liquid.^74^ However, crystalline particles have been observed also outside the expanding bubble, preceding the bubble's expansion front, by small‐angle X‐ray scattering (SAXS) experiments,^75^ confirming the theoretical predictions made by Zhigilei et al.^76^ Due to the high amount of energy accumulated in the point of bubble collapse, rebound cavitation bubbles can grow and collapse again, depending on system parameters such as liquid mechanical properties and initial bubble energy. The Rayleigh–Plesset equation is only suitable to describe the first oscillation, while the Gilmore model including the liquid compressibility is required for modelling the subsequent oscillations (rebounds).^77^ Note that even Gilmore model cannot adequately describe bubble dynamics with broken symmetry. In detail, the LAL bubble neither has as a perfect hemisphere aspect ratio (which dynamically strongly deviates from 0.5, in particular at expansion and collapse phase) nor has a circular contour. The bubble contour's circularity is broken at the interface layer directly on top of the target, so a LAL bubble geometry can—simplified—be divided into the root part on which a quasi‐hemispherical cap sits. These effects become quite obvious at smaller bubble sizes (i.e., smaller LAL pulse energies) and are particularly expressed in high viscosity liquids.^78^ After bubble collapse and NP release in the liquid, NPs may further oxidize^79^ and slowly grow on a relatively limited extent or may undergo agglomeration if they have limited colloidal stability.^10^, ^18^ Importantly, the requisite of transparency at laser wavelength holds also for the laser‐generated NPs, that may reduce productivity per pulse due to absorption and scattering (and by strongly reducing the Kerr limit for optical breakdown). In fact, the ablation rate is higher when using low wavelengths as long as no NPs synthesized by previous pulses are present (Figure 4 A–C).^10^, ^80^ Besides, it was demonstrated that productivity is affected also by persistent microbubbles stemming from vaporization or even degradation of the vapour layer at the boundary with the plasma plume,^27^ so that huge amount of permanent gases are formed proportional to the redox potential of the target.^81^ It is worth noting that laser beam absorption by NPs (known as beam self‐absorption) may induce structural and chemical changes to the NPs (LFL or LML like). Structural and chemical changes are associated with broadening and polymodality of the size distribution, as well as to phase heterogeneity of products.^10^, ^18^ To avoid or limit the self‐absorption of the primary beam, infrared or near‐infrared wavelengths, and LAL with a continuous flow set up (see Figure 2), are usually preferred.

Besides, when the productivity needs to be pushed on the gram scale, sufficiently high laser fluence (Figure 4 D–F), low pulse duration (Figure 4 G–I) and high repetition rate lasers (> kHz, that is, inter‐pulse delay of <1 ms) have shown to be most successful up until now.^10^, ^80^

But, it is worth to stress that limitation to the general trends reported in Figure 4 needs to be considered. For instance, the dependence of productivity versus fluence (Figure 4 F) depicts the regime where the fluence is below ≈7.4 times (*e* ^2^) the ablation threshold found (*ϕ*_th_) to be valid for fs‐, ps‐ and ns‐laser pulses.^33^, ^82^, ^83^ When the fluence reaches *ϕ*_th_⋅*e* ^2^ the productivity was found to reach a maximum.^33^, ^82^ Here, the former was initially predicted by the theoretical model of Neuenschwander et al. linking the increase of ablation rate with the increasing optical penetration depth into the target (with rising laser fluence).^84^ At laser fluence above *ϕ*_th_⋅*e* ^2^ the optical penetration depth is limited^84^ and hence (further increasing) only leads to a stagnating or even decreasing productivity.^33^, ^82^ In this context, self‐focusing and filamentation effects in the liquid medium, occurring especially for high laser intensities (>10^13^ W cm^−2^,^56^ usually only reached with ultra‐short pulses such as fs up to several ps), may further decrease the productivity at high laser fluence as significantly less energy reaches the target in this case.^82^

In general, the laser fluence can be increased by varying the pulse energy of the laser (Figure 4 D) or decreasing the beam spot size by optimizing the working distance between the target and focusing lens (Figure 4 E). Again note that, if the spot size becomes too small, the productivity will drop,^10^, ^80^ since the penetration depth of light into the matter is limited by the material's absorption properties (temperature‐dependent reflectivity). In other words, a linear increase in fluence is not compensated by a linear increase in the ablation efficiency.^10^, ^85^ Consequently, increasing the laser fluence by lowering the spot size from the mm scale to the micron scale will lead to a smaller ablation rate (Figure 4 F).^10^, ^80^ Additionally, especially from the work of Kautek and co‐workers,^86^ the beneficial effect of material defects in lowering the ablation threshold is known for decades. With a smaller spot size, generally less defects would interact with the laser pulse such that a higher ablation threshold (and thereby a lower productivity) can be expected with decreasing spot size.^86^

In case of pulse duration, two processes need to be considered for productivity: 1) thermal loss, which is highest in case of longer pulse duration (>10 ps, see Figure 4 G) and 2) plasma shielding (Figure 4 H) which is especially pronounced for ns laser pulse duration and above (in line with plasma dynamics, compare Figure 4 I and Figure 2).^10^, ^80^ Hence, highest ablation efficiency is usually predicted in case of fs‐ and ps‐ laser ablation as depicted in Figure 4 I.^10^, ^80^ While cavitation bubble shielding was neglected by employing sufficiently optimized scanning speed in both cases, the productivity, therefore, seems not only to decrease with increasing pulse duration but it appears that there exists an additional sweet spot of pulse duration, material and pulse energy.

Hence, at the same nominal fluence (the effective fluence decreases with increased pulse duration) there will be a material‐specific optimal laser pulse duration that also avoids losses by NP absorption, for example, 2 ps for LAL of gold.^87^ Above the kHz frequency threshold, the lifetime of the cavitation bubble exceeds the inter‐pulse delay (Figure 4 J–L), so that the laser beam propagates through a highly scattering liquid/gas interface that is responsible for a sensible reduction in the ablation efficiency. When the inter‐pulse distance is too short, the subsequent laser pulse and the cavitation bubble generated from the previous pulse overlap in time (Figure 4 J). In this case, extensive scattering of the laser pulse occurs and, hence, the productivity decreases (Figure 4 L).^10^, ^80^ These issues were solved by Barcikowski's group using a polygon scanner (compare Figure 2) coupled with a single galvanometric mirror, allowing the laser beam to spatially bypass the cavitation bubble (Figure 4 K) at MHz repetition rate and laser power ≈500 W (ps‐laser). The polygon scanner allows supersonic scan‐rates and ablates a different position of the target with each pulse.^33^, ^41^ Ablation rates of several grams per hour were reached with this high‐end class laser type.^83^, ^88^ Interestingly, Dittrich et al. have shown that the ablation efficiency (but not the absolute productivity) of ultra‐low‐power and comparably cheap compact ns‐lasers is a factor of 8 higher compared to the high‐end class ps‐laser.^83^

#### Mechanistic insights

The processes involved in laser ablation of solids in liquid environment have been the subject of extensive studies,^86^ with a special focus on non‐thermal processes and the early stages,^26^, ^89^ usually with metallic targets and ultrashort pulses. Many efforts were made to develop in situ characterization methods and numerical simulations, leading to the conclusion that the processes occurring at the early time scales after laser energy deposition are critical in the definition of the final product.^26^

Figure 3 also lists the different characterization methods reported in the literature with the reachable time scale for each of them. These measurements gave evidence on the early generation of the NPs. Light scattering experiments,^90^, ^91^ as well as in situ time‐resolved small‐angle X‐ray scattering (SAXS),^75^, ^92^ not only showed that the NPs are confined inside the vapor bubble but also showed that NPs are present earlier than bubble formation. In particular, light scattering experiments suggest that NPs are present after a few hundreds of nanoseconds.^90^, ^91^ This is consistent with the fast cooling of the plasma reported from plasma spectroscopy (10 K ns^−1^)^60^ or depicted in modelling.^22^, ^93^, ^94^

**Figure 5:**
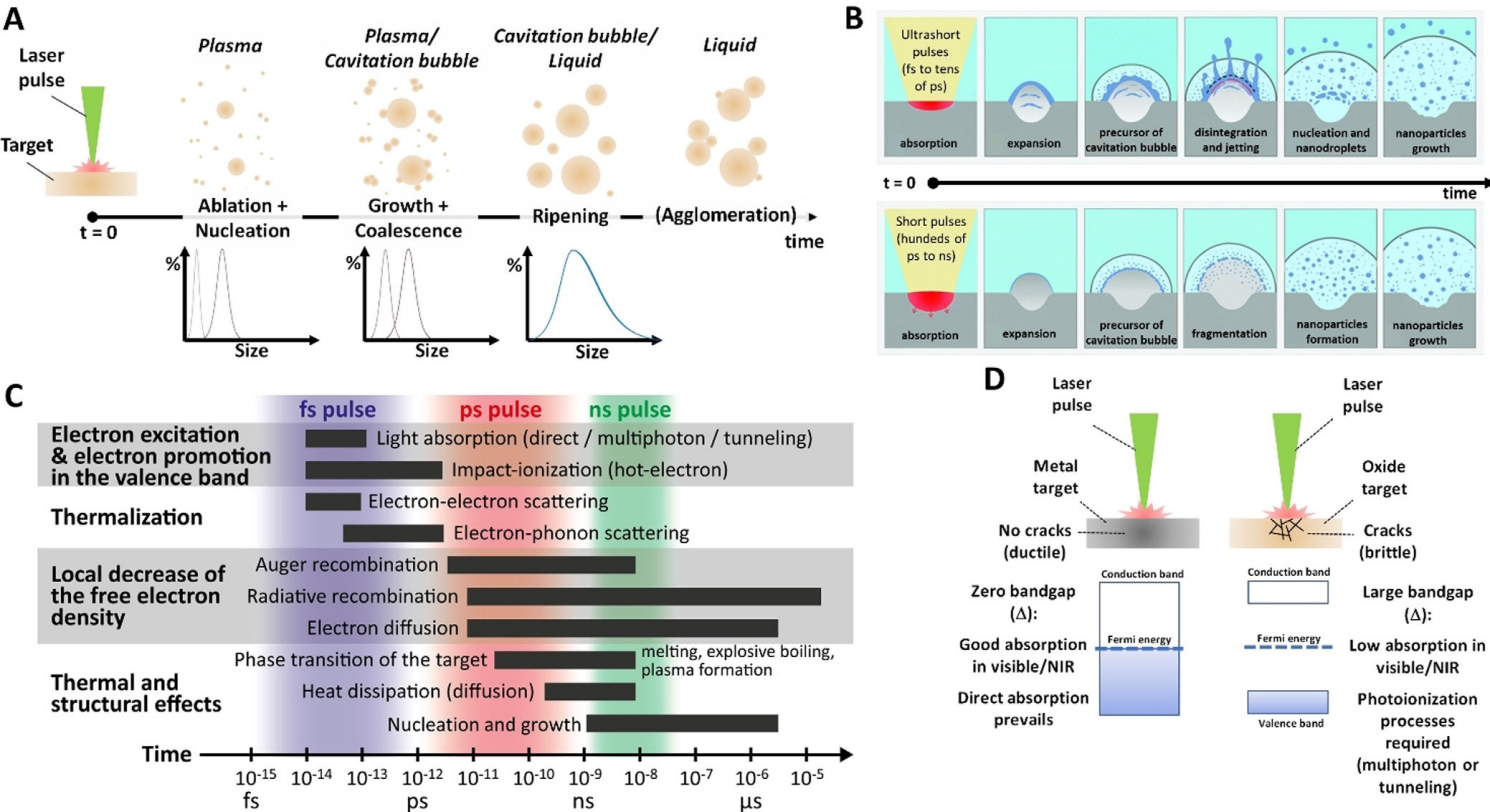
(A) Sketch of NPs formation through ablation (laser‐induced jetting) of the target material (that in general may happen as fragments and large droplets), growth and coalescence, ripening and, eventually, agglomeration if the system is not colloidally stable. (B) Sketch of the processes initiated by irradiation of a metal target in water by an ultrashort (top, femtosecond to tens of picosecond) or short (bottom, hundreds of picoseconds to nanoseconds) laser pulses. Blue: liquid; grey: metal target; light grey: ablation plume; light blue: cavitation bubble precursor; dark blue: ejected materials. For description see text. Republished with permission from ref. 22, Copyright 2020, Royal Society of Chemistry. (C) Timescales of various electron and lattice processes in laser‐excited solids.^89^ Each bar represents an approximate range of characteristic times consistent for carrier densities from 10^17^ to 10^22^ cm^−3^. (D) Sketch of main differences in laser ablation of metallic and oxide targets.

The fast cooling of a laser‐generated hot gas or plasma commonly leads to nucleation and growth of particles.^53^, ^95^, ^96^ The standard pathway to the formation of nanoparticles generally includes three stages (Figure 5 A): nucleation, evolution of nuclei into seeds, and seed growth into final nanocrystals. Although a general picture of how these steps evolve in LAL is still under construction, Zhang and Liang argued that the time required to reach the critical concentration for nucleation in LAL is much shorter than that of the wet‐chemistry synthetic routes, due to the fast ejection dynamics of the „precursors“,^53^ and related large temperature gradients of up to 10^12^ K s^−1^. Moreover, as a large part of the process takes place in the gas phase (cavitation bubble), or at relatively low concentration once in the liquid phase, where particles have slow mobility, particle growth by coalescence and ripening may last for a longer time than in the conventional La Mer mechanism used for wet‐chemistry methods.^53^ Some relevant open points for oxide nanoparticle formation concern the chemistry that is discussed in the next paragraphs. This echoes the questions of the physicochemical interaction which must be addressed to understand the fast vaporization of the solvent, as well as the parameters favouring its decomposition and its reactivity.

The scenario of Figure 5 A is supported by molecular dynamics simulations developed by Zhigilei's group.^22^, ^76^, ^93^, ^94^ They have developed atomistic simulations of the laser ablation of metal targets in water, combining a coarse‐grained representation of the liquid environment and an atomistic description of the laser interaction with metal targets and for pulse durations from fs up to few ns.^22^, ^76^, ^93^, ^94^ For ultrashort laser pulse durations a bimodal size distribution is predicted, as frequently reported from transmission electron microscopy,^97^ and also from SAXS measurements.^74^, ^75^, ^92^

In the case of hundreds of ps to ns pulses, the thermal and stress confinement characteristic of ultrashort pulse laser ablation is not observed.^22^ This is associated with different ablation dynamics and partly explains why the nanoparticle size distribution tends to be broad in ns LAL but not bimodal as in ultrashort‐pulsed LAL, as described in Figure 5 B.^22^ At the same effective fluence, independent of the pulse duration (ps or ns), three NP formation pathways are predicted by the works of the Zhigilei group, which are linked to the regions where they originate from, summarized as follows along three regional sections, from the target towards the liquid: 1) The nanoparticles mostly emerge from the spinodal decomposition of a part of the ablated material located between the target surface and the transient interfacial metal layer. High density of ablated matter (and low amount of supercritical water) is characteristic for this region. High temperature causes the seeds (1‐4 nm) to be thermodynamically unstable and therefore evaporate unless their rapid collision and coalescence leads to small (about 5 nm) nanoparticles, while the larger nanoparticles in this region continues to grow. These processes result in thermodynamically stable, mostly larger nanoparticles of around 5–10 nm, although some smaller ones survived the growth process. 2) The decomposition of the thin transient metal layer at the interface between the ablation plume and water environment causes the generation of large (>10 nm) molten nanoparticles, only slowly cooled by surrounding supercritical water. 3) At the very front of the emerging cavitation bubble, evaporation from the hot interfacial layer into the supercritical water causes the formation of very small (<5 nm) nanoparticles through the nucleation and growth from the vapor‐phase metal atoms. These small nanoparticles solidify in nanoseconds, likely in defect‐rich nanocrystals, whereas particles stemming from pathways 1) and 2) are still in molten stage after a few nanoseconds. In Figure 5 B), one obvious difference that shorter pulse duration causes is jetting of particles stemming from the pathway 2) as a result of a more vigorous ablation process, which includes the ejection of metal droplets directly into the high‐density water region. Note that the intriguing sketches in Figure 5 B) are intended to explain the mechanism^22^ but they are not to scale at all. In reality, after some ns the height of this early formation volume is only hundreds of nm, whereas the width is the laser spot size (tens of microns), so the virtual picture one should have in mind is a very flat object with an aspect ratio of about 100.

Noteworthy, there are several additional complexities when considering oxide targets instead of metal ones. First, there is a lack of optimized empirical potentials for metal oxides which could be effectively used in a molecular dynamics simulation.^98^ The reasons are the increased complexity of the interatomic potentials when several chemical elements are involved (at least the oxygen and a metallic element), and the polymorphism issue since the metal oxides usually form various stoichiometries and crystal structures. Second, while in metals there is a direct laser‐heating of the free electrons in the conduction band, in metal oxides electrons must be promoted across the band gap by the laser excitation before their heating (as discussed below). The modelling of the laser‐target interaction is then more challenging than for metals. Third, molecular dynamics are not suited yet to catch the chemistry in the early ablation phase leading to metal oxide NPs. Fourth, numerical simulation assuming ultrashort pulse duration cannot catch the whole complexity of the processes resulting from the plasma‐laser interaction which occurs for (several) nanosecond pulse duration, although recently a convincing modelling approach in that direction has been presented.^22^

Indeed, for nanosecond pulses, shielding of the target by the plasma occurs, with an increased effect with the decreasing wavelength.^99^ In addition, the surface structure such as its roughness changes significantly also on the ns time scale, which is expected to locally modify the optical properties of the target and, thus, its light absorption properties.^22^ Therefore, the energy deposited on the target, as well as plasma warming are difficult to account quantitatively.

Despite the apparent complexity of the processes, some evidence comes from the experimental investigation of the ablation processes, including ablation of semiconductors and dielectrics. Figure 5 C shows the timescales of various electron and lattice processes in laser‐excited solids for an ultrashort pulse (femtosecond). The subsequent typical pathways of energy dissipation and phase transformations following the excitation by an ultrashort pulse are also displayed in Figure 5 C. As anticipated above, the main difference between metals and metal oxides lies in the mechanisms of electron excitation due to the large energy band gap of the latter, leading to several differences in linear and nonlinear absorption (especially at NIR wavelength), as well as in electron dynamics. Overall, the ablation of metal targets could appear more convenient, which justifies why most works dealing with the synthesis of metal oxides are based on the oxidative laser ablation of bulk metals (see next paragraph). Another reason for this choice is related to the mechanical properties of the materials, since pure metals are commonly more ductile than their oxides. Brittle metal oxides are more subject to shockwave‐induced damage, which leads to target‐crushing and target break‐up (Figure 5 D). As a consequence, the mass of NPs produced by laser ablation of bulk oxide targets can strongly differ from the mass removal from the target, requiring additional purification steps to remove the unwanted target pieces. For instance, the ablation of a Gd_2_O_3_ target with an Nd:YAG laser source (500 ps, 2 mJ/pulse, 1064 nm, 2×10^11^ W cm^−2^) led to the production of NPs (diameter <100 nm) of 2.00±0.18 ng/pulse, which however corresponds to only 13 % of the removed material from the target (≈15 ng/pulse).^100^ In this context, the porosity of pressed YIG (yttrium iron garnet) micro‐powder targets was shown to be detrimental, since a high porosity leads to a large fraction of microparticles detaching from the target and being present in the colloid.^101^ Conversely, the ablation of a high‐density powder target (>99 %) led to the formation of ≈3 nm YIG NPs similar to the ablation of a bulk YIG target.^101^

Concerning the ablation mechanism of oxide targets with common laser sources used in LAL, such as the 1064 nm nanosecond pulses of Nd:YAG laser in fundamental mode, the photon energy (1.17 eV) is lower than the band gap energy of most oxides. As an example, the energy of 8 photons is needed to cross the Al_2_O_3_ band gap with a 1064 nm laser source. The promotion of the electrons from the valence band thus involves photoionization processes preceding avalanche ionization. Photoionization acts as an initial step in the laser energy deposition and subsequent material modifications, leading to the optical breakdown of the solid.^102^ There are two different processes of photoionization, multiphoton ionization and tunnelling ionization.^103^ The multiphoton excitation involves the simultaneous absorption of several photons. The ionization by electron tunnelling is induced by a distortion of the potential barrier for large laser fields, and it is relevant for high electric field intensities, as those reached with ultrashort laser pulses.

A better understanding of the ablation conditions is provided by the value of the parameter *γ*, which gives the balance between the multiphoton ionization regime and the tunnelling ionization regime and is defined as [Eq. (1)]:

(1) $$\gamma =\frac{2\pi c}{\lambda }\frac{\sqrt{m\Delta }}{eE}$$

where *λ* is the laser wavelength, *c* light speed, *m* the reduced mass of the electron, *e* the electron charge, Δ the energy band gap of the material, and *E* the magnitude of the laser electric field, which scales with $\sqrt{1/\tau }$ , where *τ* is the pulse duration.^104^ When is decreased below 1, the optical breakdown is governed by the tunnelling ionization regime,^105^ that is regarded as a „deterministic breakdown regime“, that is, with high precision in the spatial delivery of pulse energy to the target.^103^, ^106^ According to Equation (1), decreasing γ to reach a deterministic breakdown regime can be achieved by increasing the wavelength and decreasing the pulse duration.

Once electrons are promoted to the conduction band, they can absorb laser energy through inverse bremsstrahlung (electron acceleration). For ns pulses at ordinary fluencies, electron acceleration is equilibrated by electron‐phonon scattering. Conversely, for ultrashort pulses, electrons are accelerated up to the threshold for achieving avalanche multiplication by electron‐electron scattering, thus further increasing the electron density in the conduction band.

For semiconductors, one can imagine that the initial presence of free carriers (doping) could help to decrease the ablation threshold. For dielectrics, optical defects could also help to decrease the ablation threshold. However, Leyder et al. have compared the non‐linear absorption inside silicon for samples with different initial free‐carrier densities,^107^ that is, for doping concentrations from 10^13^ cm^−3^ to 10^18^ cm^−3^. For a 130 fs pulse at 1.3 μm, their results demonstrate that the laser energy deposition does not depend on the doping concentration, and thus the avalanche is not efficiently triggered even up to a 10^18^ cm^−3^ free electron density. The multiphoton excitation is a nonlinear process highly sensitive to the laser intensity *I*, leading to a scaling law *I* ^*N*^ for the absorption with *N* the number of photons involved.^108^ The ablation threshold is decreased at a shorter wavelength, with a consequent increase in the ablation rate.^108^, ^109^, ^110^

To increase LAL efficiency with oxide targets, it could appear convenient to use short wavelengths in the near UV, but with the drawback of self‐absorption of the laser beam by the produced NPs (see Figure 3 B–D). Another approach to improve the ablation efficiency relies on promoting material breakdown in the tunnelling ionization (deterministic) regime instead of the multiphoton one.

On the other hand, the decrease in pulse duration is associated with the issue of self‐focusing and liquid breakdown or filamentation for such high fluences (for instance the threshold of optical breakdown in water is 1.11×10^13^ W cm^−2^).^111^ Recently, Doñate‐Buendía et al. have elegantly overcome the issue of the filamentation and non‐linear energy losses in the water when femtosecond laser sources are used in LAL.^55^ They have applied the simultaneous spatial and temporal focusing (SSTF) of femtosecond pulses configuration, that avoids the unwanted non‐linear effects, and ensures a thigh control of the ablation spot for a femtosecond laser source (45 fs pulse duration, 800 nm, 1 kHz, 200 μJ/pulse, 7×10^13^ W cm^−2^ at the focal point in water).^55^ Though promising, the best productivities to date remain those obtained with high repetition rate ns and ps laser sources coupled with scanning optics to bypass the cavitation bubble.^33^, ^112^

### Overview of oxide NPs obtained by LAL

#### Conventional nano‐oxides: role of bulk target.

**Figure 6:**
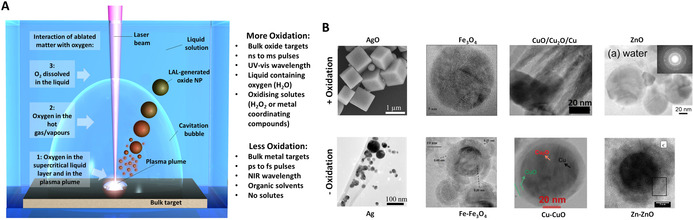
A) Sketch of LAL highlighting the three environments where the target species encounter oxygen species: in the plasma plume (1), in the cavitation bubble (2) and in the liquid at ambient conditions (3). B) By changing the LAL parameters listed in (A), it is possible to switch from AgO microcubes to metal Ag (adapted with permission from ref. 123, Copyright 2011, American Chemical Society, from Fe_3_O_4_ (adapted with permission from ref. 119, Copyright 2011, Royal Society of Chemistry), to Fe‐Fe_3_O_4_ (reprinted with permission from ref. 157, Copyright 2011, American Chemical Society), from CuO/Cu_2_O/Cu (reprinted with permission from ref. 180), to Cu‐CuO (reprinted with permission from ref. 173, Copyright 2019, Elsevier), or from ZnO (adapted with permission from ref. 144, Copyright 2005, American Chemical Society) to Zn‐ZnO NPs (adapted with permission from ref. 142, Copyright 2005, American Chemical Society).

The working principle of LAL seems to suggest that the easiest way to produce oxide NPs is starting from a bulk oxide target. However, this is the less frequent case found in the literature, where the majority of works report the laser ablation synthesis in liquid solution of oxide NPs starting from a bulk target of pure metal. In fact, it is worth noting that in LAL, the matter extracted from the solid target ineluctably encounters the molecules of the liquid solution, in three different conditions which are (Figure 6 A): 1) interface between the ablation plume and the surrounding liquid, 2) the interior of the cavitation bubble, in the gas phase and 3) the liquid at ambient temperature and pressure after the collapse of the cavitation bubble. The formation of radical species during laser‐induced breakdown of solvent molecules in the plasma at target surface has been intensively investigated in the last years,^27^, ^113^, ^114^, ^115^ showing that these radicals may react forming persistent microbubbles consisting amongst others of H_2_, O_2_,^27^, ^115^ and H_2_O_2_ 114 when LAL is performed in water. Excited oxygen species have been observed in real‐time inside the plasma plume in aqueous environment, up to hundreds of nanoseconds after pulse absorption,^116^ and also in ambient air during ablation of oxide targets.^117^ Hence, oxygen coming from the molecules of liquid (e.g., H_2_O) or additives (e.g., H_2_O_2_ or atmospheric O_2_) will react with the ablated target species, and the extent of the oxidation reaction will depend on the type and concentration of reactive oxygen species and on the redox potential of the metal.^81^ This is the source of persistent microbubbles affecting the ablation rate.^27^ For the ablation of 7 different metals, Kalus et al. observed that the developed gas volume is directly correlated with the respective redox potential of the metal.^81^ A possible correlation of (temperature‐dependent) redox potential and oxidation state during LAL has been discussed recently in literature.^19^

All this makes the choice of the bulk target crucial for the achievement of the desired oxide NPs. The case of iron nano‐oxides is useful to exemplify this aspect, given the variety of possible iron compounds and the relatively simple characterization.^118^, ^119^ It has been reported that ns‐laser ablation of bulk metal Fe target in water gives a prevalence of magnetite Fe_3_O_4_ NPs, with a minority of hematite α‐Fe_2_O_3_, wustite FeO and even some traces of metal Fe, likely present as a core inside a protecting oxide shell.^118^ However, when ns‐laser ablation is performed with a hematite target in water, amorphous Fe_2_O_3_ particles are collected.^120^ Conversely, maghemite (γ‐Fe_2_O_3_) nano‐oxides are obtained from the hematite target in ethanol or acetone, which are known to have a partially reducing effect on the ablated material during LAL.^69^, ^121^ This is in agreement with computer simulations and experiments performed on alumina, indicating that a slight excess of oxygen is required to achieve oxide NPs with the same stoichiometry of the target when ablated material is still in the gas phase of the cavitation bubble.^117^

This suggests that LAL of oxide targets in organic solvents such as alcohols or acetone give slightly oxidized NPs, while oxidation is promoted in water eventually forming amorphous and hydroxylated compounds. Nonetheless, crystalline oxide NPs can be obtained by laser ablation of crystalline oxide targets in water, as demonstrated with 1064 nm (13 ns) pulses and a 98 wt.% ZnO: 2 wt.% Al_2_O_3_ target in MilliQ water.^122^ Conversely, LAL of metal targets in aqueous environments gives oxides, sub‐stoichiometric‐oxides or hybrid metal‐oxide structures. These cases are discussed in better detail and with the help of specific examples in the next paragraphs.

#### Conventional nano‐oxides: role of oxide type and LAL parameters

In several cases, LAL allows tuning the composition and the structure of oxide NPs, ranging from compounds where the metal has the largest possible oxidation state, to core–shell structures where only an external shell of oxide is formed around a core of pure metal (Figure 6 B). The presence of oxygen atoms in the plasma plume is associated to highly oxidative conditions, such that even noble metal (Ag,^123^ Au,^124^, ^125^ Pt,^126^ Pd,^127^ Rh^128^, ^129^) NPs with a fraction of oxidised atoms have been observed. Oxidation is promoted by the use of ns laser pulses, UV wavelength and ion stabilizing additives, compared to pulses with a shorter duration, use of NIR wavelength, or presence of additives not interacting with metal ions. For instance, Ag_2_O nanocubes were obtained with 248 nm (30 ns) LAL of bulk Ag in aqueous solutions of polysorbate, while metal Ag NPs with limited surface oxidation are obtained in similar conditions without adding polysorbate,^123^ or at 1064 and 532 nm in aqueous solutions of sodium dodecyl sulphate.^130^ UV light is re‐absorbed by the NPs, giving a simultaneous process of LAL and LFL, with the result of increasing the chance of reaction between metal and oxygen atoms. The role of the ion stabilizing additive is that of slowing down the rate of electron transfer from reducing species to coordinated metal cations in solution, thus increasing the probability of reaction with oxygen.^123^

Chemical oxidants can be added to the solution to promote the reaction of target atoms with oxygen, as shown by LAL of bulk Cu in pure water and aqueous solutions of H_2_O_2_ (1–5 vol.%) with 532 nm (5 ns) laser pulses, obtaining, respectively Cu_2_O or CuO nanocrystals.^131^ LAL with 355 nm ns pulses in 3 vol.% H_2_O_2_ has been reported also with a Ni target to achieve NiO NPs.^132^ In another report, gallium oxide Ga_2_O_3_ has been found after LAL of pure GaAs in acetone with 532 nm (7 ns), while non‐oxidised GaAs NPs were achieved when using 250 fs pulses in the same conditions.^133^ LAL with 1064 nm (10 ns) pulses of a GaN target in water also originated GaNO NPs.^134^ Regarding the pulse duration, the different results may be ascribed to the longer lifetime of the plasma plume when longer laser pulses are used. This is associated with a more extended mixing of target and solution species in the highly reactive plasma conditions,^10^, ^18^, ^26^ as well as in a longer lifetime of the cavitation bubble and potentially prolonged persistence of NPs in the gaseous phase at temperature ≫ than room temperature.^26^, ^77^, ^135^, ^136^ In fact, there are many reports where LAL with ns pulses of metal targets in water produced oxide NPs. For instance, γ‐Al_2_O_3_ nanocrystals co‐doped with H^+^ and Al^2+^,^137^, ^138^ Co_3_O_4_,^139^ Fe_3_O_4_,^118^, ^119^ TiO_2_ 29 and MoO_3_ 140 NPs were produced by 1064 nm ns pulses starting from a target of, respectively, metal Al, Co, Fe or Mo in water. Wurtzite ZnO NPs were produced with 1064 or 532 nm ns pulses starting from a Zn target in water.^141^, ^142^, ^143^, ^144^ Photoluminescence measurements showed that ZnO particles obtained by LAL can be rich with oxygen vacancies.^145^ ZnO particles co‐doped with Al_2_O_3_ have been synthesized starting from a bulk ZnO target doped with 2 wt. % of alumina, dipped in water, and using 1064 nm ns pulses.^122^ Recently, non‐oxidized Zn atoms from a Zn target ablated in water with 7 ns–1064 nm pulses were detected inside the cavitation bubble still after tens of microseconds by in situ x‐ray absorption spectroscopy, and the metal signature prevailed even for milliseconds (i.e. after bubble collapse).^51^

SnO_2_ was produced by 355 nm (10 ns) LAL of Sn in water.^146^ Analogous results were reported when using longer laser pulses of 6 μs at 1064 nm and a target of Gd in diethylene glycol, which produced Gd_2_O_3_ NPs by the reaction of ablated Gd atoms with atmospheric oxygen dissolved in the liquid and oxygen coming from solvent pyrolysis.^147^ The use of laser pulses with longer duration is likely to further extend the plasma lifetime and volume compared to ns‐pulses, facilitating the ionization of solvent molecules and the reaction of target and solution species in the plasma or nearly plasma conditions.^26^, ^77^, ^135^ Interestingly, the average size of Gd_2_O_3_ NPs is 4 nm, significantly lower than the average size of oxide NPs obtained by LAL in water (at the same pulse duration of 6 μs), that ranged between 10 and 30 nm.^118^, ^119^, ^137^, ^138^, ^139^ This is attributed to ethylene glycol properties, such as adsorption on the surface of Gd oxide clusters and high viscosity hindering cluster coalescence.^18^, ^147^ Oxidation of target species occurs also during LAL of organic materials like coal in ethanol and 355 nm (10 ns) pulses, giving graphene oxide quantum dots.^148^

When LAL is applied to oxide target, the surrounding liquid may influence the crystallinity of final products. This has been shown especially for the LASiS of rare‐earth‐doped oxides, such as YVO_4_:Eu^3+^ NPs obtained with 532 nm (10 ns) pulses in water, ethanol or mixtures of the two liquids.^149^ The different interaction of solvent molecules with the surface of YVO_4_:Eu^3+^ NPs was evident from the achievement of crystalline ovoidal particles in water versus spherical and partially amorphous particles in presence of ethanol,^149^ or spherical crystalline particles in an aqueous solution of SDS.^150^ This suggested that YVO_4_:Eu^3+^ nanocrystals are stabilized more effectively by ethanol molecules than by aqueous surfactants like SDS. Indeed, the reactivity of organic solvents with the ablated species needs to be considered for each specific material and set of synthetic parameters. For instance, LASiS of GaO colloids has been reported with 1064 nm (10 ns) pulses and a GaO target in ethanol,^151^ or defective CeO_2_ nanocrystals were produced by 1064 nm (10 ns) pulses and a CeO_2_ target in water.^152^ Instead, in the case of Ti target and 1064 or 532 nm ns pulses, the oxide phases or rutile and anatase TiO_2_ were achieved only in water, while TiC was found in alcohols.^153^, ^154^, ^155^, ^156^ Also in the case of Fe targets and 1064 nm (10 ns) pulses, magnetite (Fe_3_O_4_) was found in solvents like acetonitrile and dimethylformamide, while a mixture of magnetite and iron carbide (Fe_3_C) was obtained in ethanol.^157^ Analogous results were found in LAL of Fe with fs pulses.^158^, ^159^

In some cases, the reactive oxygen species generated in the plasma plume from water molecules may interact strongly with some metals. For instance, W poly‐oxo‐clusters were obtained with 1064 nm (10 ns) pulses and W target in water. The stabilization of poly‐oxo‐clusters is possible also by the addition of strongly interacting ligands to the liquid solution, as demonstrated with 1064 nm (6 ns) LAL of iron in aqueous solution of *N*‐(phosphonomethyl) iminodiacetic acid (PMIDA), which generated nanoaggregates of iron poly‐oxo‐clusters.^160^

#### Core‐shell NPs

When LAL is performed in organic solvents under inert (Ar, N_2_) atmosphere, even the elements with the highest tendency to oxidation and featuring a non‐passivating oxide (such as Fe) may be obtained stable in liquid with a pure metal core protected by an oxide layer.^157^, ^161^ Such metal‐oxide core–shell NPs are formed when the concentration of reactive oxygen species in the plasma plume is low enough to avoid the complete oxidation of the metallic core. Such a condition occurs in organic solvents without oxygen in their chemical formula. Nonetheless, dissolved oxygen is present in the liquid at equilibrium with the ambient atmosphere, then oxygen atoms are present inside the cavitation bubble and in the liquid phase, causing to the oxidation of metal atoms in the outer shell of the laser‐generated NPs (Figure 6 A). The formation of core–shell NPs is observed also in solvents containing oxygen in their chemical formula, like alcohols or tetrahydrofuran,^121^ because oxygen atoms are sequestrated by oxygen‐scavenging species from the degradation of solvent molecules in the plasma plume, reducing the overall amount of oxygen. The amount of oxygen can be controlled also introducing scavenging species in the target, such as a sacrificial layer of iron in an iron‐gold thin bilayer film.^113^

There is a conspicuous list of core–shell NPs obtained in one step by LAL. For instance, LASiS of NPs composed of a Fe core and an iron oxide shell has been demonstrated in tetrahydrofuran,^157^ acetone,^162^ and alcohols.^163^, ^164^ Particles of metal Ni core with nickel oxide shell have been produced by LAL of Ni target in water or alcohols.^28^, ^165^ Zn‐ZnO core–shell NPs were obtained by 1064 nm LAL in an aqueous solution of SDS.^142^ LAL with 1064 nm (7 ns^166^ or 30 ps^167^) pulses and Mo target in water formed NPs with a metal Mo core surrounded by an oxide‐hydroxide Mo shell, with shell thickness increasing with the ageing time of the solution. Si NPs passivated by a SiO_2_ shell were produced by LAL of Si in water or organic solvents.^168^, ^169^ Fe‐FeMn_2_O_4_ NPs were obtained by laser ablation of a FeMn target in ethanol with 1064 nm (10 ps) pulses.^170^ In the case of a FeNi target in acetone and ps pulses, NPs with a core of mixed Fe‐Ni carbide and a shell of iron oxide were obtained.^171^ Formation of iron oxide shell was observed also during LAL with 1064 nm (6 ns) pulses of Au‐Fe alloys^161^ and Au/Fe bilayer thin film targets in water.^113^ Cu‐Cu_2_O core–shell NPs have been obtained in methanol and 2‐propanol, with shell thickness increasing with ageing time.^172^ In some cases, Cu‐Cu_2_O core–shell NPs have been observed also after LAL of Cu in water with 532 or 1064 nm ns pulses.^173^, ^174^, ^175^ Hollow cobalt oxide nanospheres were observed during LAL of Co in water, due to the Kirkendall effect.^132^, ^176^ In the case of 1064 nm ns LAL of Ta in ethanol, NPs resulted in a core of partially oxidised Ta coated with a shell of Ta_2_O_5_.^177^ Also, oxocarbon‐encapsulated metal^178^ or oxide NPs^179^ have been obtained by LAL, exploiting the pyrolysis of organic solvents inside the plasma plume.

#### Ligand‐stabilized oxide NPs

Addition of ligands or other solutes to the liquid before LAL can help to achieve better control of the colloidal stability, as well downsizing the produced NPs. Organic ligands or inorganic compounds (e.g., salts) are usually used.^10^ The aggregation of the NPs is avoided due to steric or electro‐steric hindrance when using organic ligands, while salts improved the electrostatic repulsion by increasing the surface charge of the NPs. Such a positive effect of the ligands has been reported for both laser ablation in liquids (LAL) and laser fragmentation in liquids (LFL).^10^ Although these methods are commonly used in colloidal chemistry, the first systematic use of ligands in the framework of LAL has been reported in the seminal studies of Mafuné et al. for noble metal particles.^130^, ^181^ They demonstrated a shift of the NPs size distribution toward smaller sizes when the concentration of the ligand is increased. The authors used an aqueous solution of sodium dodecyl sulfate (SDS), an anionic surfactant, for the narrowed‐size synthesis of silver NPs^130^, ^181^ and gold NPs.^182^ Thereafter, the same method has been applied for the preparation of metal oxide nanomaterials, such as SnO_2_, TiO_2_, and YVO_4_:Eu^3+^ in SDS aqueous solutions.^146^, ^150^ Moreover, the crystallinity of the NPs and their abundance seem to strongly depend on the SDS concentration. Usui et al. succeeded in preparing ZnO NPs by laser ablation of a zinc metal plate in solutions of deionized water with different surfactants like lauryl dimethylamino acetic acid betaine (LDA), cetyltrimethylammonium bromide (CTAB), and octaethylene glycol monododecyl ether (OGM).^144^

**Figure 7:**
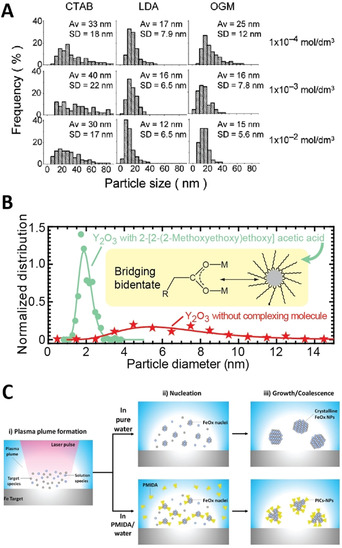
(A) Size distributions of ZnO NPs prepared in CTAB, LDA, and OGM surfactant solutions with various concentrations. Av and SD represent the average and standard deviation of the corresponding particle size distribution (reprinted with permission from ref. 144, Copyright 2005, American Chemical Society). (B) Shift and sharpening of the size distribution when 2‐[2‐(2‐methoxyethoxy)ethoxy]acetic acid is used to produced Y_2_O_3_ NPs (reprinted with permission from ref. 63, Copyright 2011, American Chemical Society) (C) Simplified sketch of the mechanism of phosphonate‐grafted iron oxo‐clusters formation. In pure water, crystalline FeO_*x*_ NPs are obtained. When *N*‐(phosphonomethyl) iminodiacetic acid (PMIDA) ligands are present, PMIDA can coordinate iron oxo‐clusters already in the early stage of FeO_*x*_ nucleation. Consequently, the formation of crystalline FeO_*x*_ phases is not possible (as illustrated in the bottom part of the panel; Reprinted with permission from ref. 160, Copyright 2018, Elsevier).

Figure 7 A shows that different size distributions of the ZnO NPs are found by varying the type and concentration of the three surfactants. The carboxylate (R‐COO^−^) group and phosphonates are also well known to react with mineral surfaces, with various coordination modes to metal ions. The average size of Y_2_O_3_ NPs dramatically shifts from above 6.4 to 1.9 nm in a solution of 2‐[2‐(2‐methoxyethoxy)ethoxy]acetic acid (Figure 7 B).^63^ The same ligand has been used to stabilize lanthanide sesquioxides and yttrium aluminium garnet (YAG),^63^ but also upconversion NPs NaYF_4_:Yb,Er.^183^ *N*‐(phosphonomethyl) iminodiacetic acid (PMIDA) has been used to stabilize nanoaggregates of iron poly‐oxo‐clusters (Figure 7 C).^160^

In LAL, the drastic size quenching observed with ligands^63^, ^160^ (see Figures 6 B–C) suggests an early quenching of the particle growth, and thus an early penetration of the ligands into the environment where NPs form. This echoes the questions on the process leading to the fast vaporization of the solvent,^71^ to its decomposition, and then to its reactivity.^184^ On the one hand, there is no doubt that solvated species penetrate the plasma. Indeed, the transfer of solvated ions (Na^+^, Li^+^) has been reported using plasma spectroscopy.^64^ Laser ablation of a gadolinium oxide in a europium chloride solution leads to europium doped gadolinium oxide NPs, which demonstrates that impurities from the solvent can effectively penetrate the core of the NPs as they grow.^100^ SAXS measurements performed during laser ablation of gold in a NaCl solution show that the size quenching usually reported during laser synthesis of metallic particles in a solution of low salinity happens already in the vapor phase of the cavitation bubble.^185^ This has recently been confirmed for organic ligands.^186^ However, such penetration of solvated ions or organic molecules is unexpected for standard vaporization. It has been suggested that the water experiences explosive boiling for nanosecond laser pulses. On the other hand, one could expect a decomposition of the organic ligands in contact with the plasma, since the decomposition of the solvent itself has been reported.^187^ It would suggest that the plasma is quickly quenched by the solvent vaporization, leading to a fast decrease of the kinetic temperature and then to the nucleation of the particles, while their subsequent growth is quenched by the ligands supply by the solvent vaporization. Then organic ligands act in the vapor phase, probably early after the plasma quenching, in a more friendly environment.

The use of polymers, biopolymers, gelatin, albumin, starch, and chitosan during LAL has been also explored, although mainly on metallic particles.^10^ In the case of ZnO NPs obtained by laser ablation of a ZnO plate in starch solutions,^188^ the starch acted as a complexing template that prevented both aggregation and crystal growth through steric hindrance.

#### Multicomponent oxide nanostructures by sequential LAL or reactive LAL

**Figure 8:**
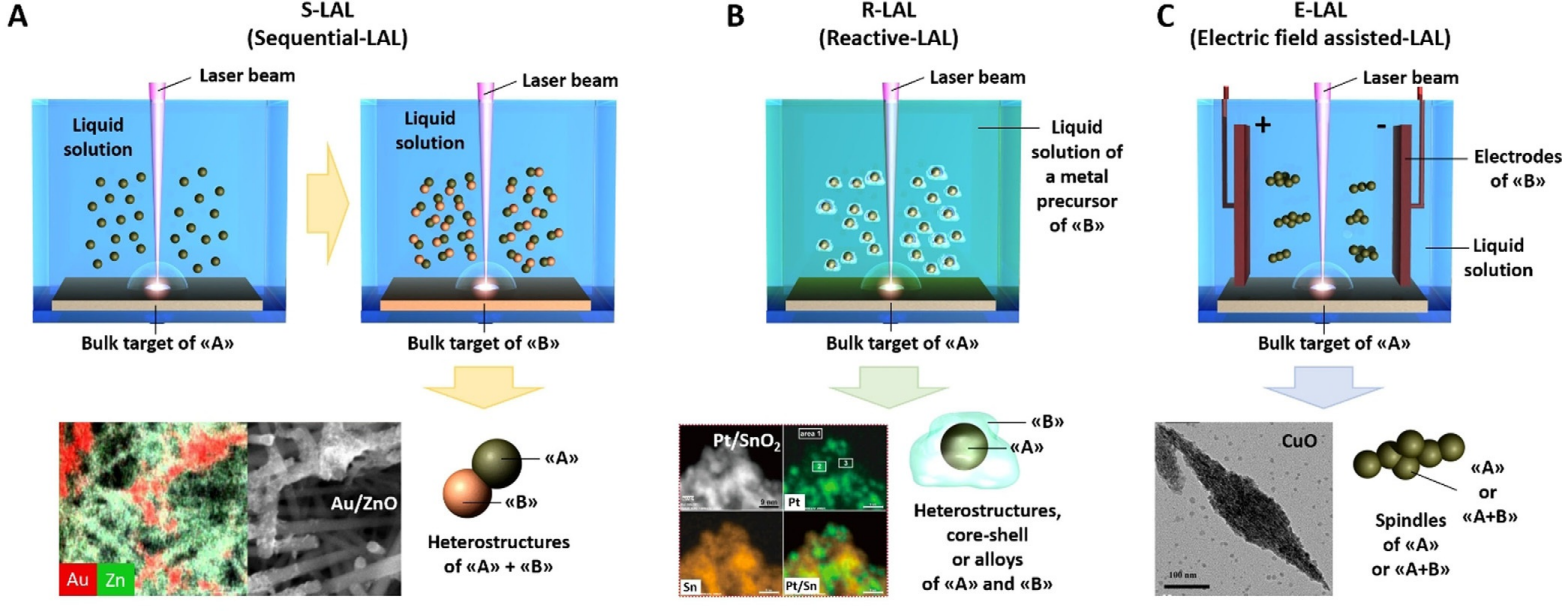
A) Sketch of S‐LAL with the targets of two distinct materials „A“ and „B“ to give multicomponent „A“+„B“ heterostructures like Au/ZnO. Adapted with permission under CC BY 4.0 from ref. 141, Copyright 2016, EDP Sciences). B) Sketch of R‐LAL with a target of a material „A“ to give NPs that interact with a chemically reactive precursor of „B“ to give a multi‐component „A/B“ system like Pt/SnO_2_ (adapted with permission from ref. 190, Copyright 2019, Elsevier). C) Sketch of E‐LAL with a target of a material „A“ in the presence of an electric potential applied by an electrode of a material „B“ to give elongated nanostructures like CuO nanospindles, or multi‐component „A“+„B“ oxides (reprinted with permission from ref. 193, Copyright 2009, American Chemical Society).

Sequential LAL (S‐LAL, Figure 8 A) has been frequently used to produce heterostructures of oxides with other nanomaterials, typically metals like Au or Pt. In S‐LAL, the laser ablation is performed sequentially on the same solution but with different targets. For instance, ZnO‐Au,^141^ and NiO_2_‐Au^189^ heterostructures were obtained by S‐LAL with Zn or Ni and Au targets. Another alternative, consisting of a single LAL step, is the addition of reactive solutes in the liquid environment before or after the laser synthesis, and it is known as reactive LAL (R‐LAL, Figure 8 B). Pt/SnO_2_, PtCo/CoO_*x*_, Au/TiO_2_, Ag/TiO_2_ and Pt/FeO_*x*_ have been obtained by adding metal salts (Na_2_PtCl_4_, K_2_PtCl_4_, HAuCl_4_, AgNO_3_) during or after the LAL of Sn, Co, Ti or Fe targets in water.^190^, ^191^, ^192^

#### Self‐assembled oxide nanostructures obtained by LAL and external electric fields or ageing

In some cases, the LAL is performed simultaneously to the application of external electric or magnetic fields.^35^ The electric field can be applied by placing two electrodes in the liquid layer above the target, or using the metal target as one of the electrodes. In the first configuration, laser ablation is performed with 532 nm (10 ns) on a Ge or Cu target in water to give, respectively, GeO_2_ or CuO nanocrystals,^193^, ^194^ where the external electric field promoted the assembly of laser‐generated NPs in anisotropic spindle structures (Figure 8 C). The same configuration has been applied to the generation of copper or silver vanadate microstructures, using 532 nm (10 ns) pulses, a V target in aqueous solutions and, respectively, Cu or Ag electrodes.^195^, ^196^ In these cases, the metal anodes are electrolyzed by the applied electric field at a suitable voltage, generating metal ions (Cu^2+^ or Ag^+^) that dissolve into the liquid environment and form hydrated species that react with laser‐generated V to form mixed metal oxides.^195^, ^196^ Therefore, the process has been defined as „electrochemical LAL“.^35^ Electrochemical LAL has been used also for the production of copper molybdate polyoxometalates (POM) in a two‐step procedure, consisting in the laser ablation of Mo in water with 532 nm (10 ns) pulses and Cu electrodes to achieve crystalline lindgrenite (Cu_3_(OH)_2_(MoO_4_)_2_), followed by annealing of the products at 500 °C for 5 h to obtain the final POM structure.^197^ Although the preparation of copper molybdate POMs required heating at high temperature, post‐LAL ageing of products at room temperature has been reported as a strategy to achieve the self‐assembly of NPs into submicron structures such as wires, leaves and flakes.^35^ For instance, Mn oxide nanocubes were formed by the ageing of Mn_2_O_3_ NPs in water, whereas nanofibers and nanosheets were observed starting from Fe‐Mn oxides.^170^ This is facilitated by the absence of stabilizing ligands or other chemical additives which typically passivate the surface of nanocrystals generated by chemical routes. In other reports, ZnO nanorods and nanoflowers were obtained instead of nanospheres by LAL of a Zn plate in water and ageing.^141^, ^198^, ^199^ The results were attributed to the heating of the liquid solution due to the long pulse duration (in case of ms pulses)^198^ and to NPs re‐irradiation (in case of 532 nm pulses).^141^, ^199^

Even when the electric potential is applied to the ablated target, the chemical composition of products can be modified, due to the alteration of redox processes between ablated species and solution species. For instance, laser ablation of Al in water with 1064 nm (10 ns) pulses generated crystalline boehmite (AlOOH) NPs when the target was biased with a negative potential of −10 V versus the counter electrode in solution, while the fraction of crystalline hydroxide is much lower when LAL is performed without any electric field.^200^

#### Issues related to compositional homogeneity

In a minority of cases, crystalline hydroxides were found together with oxide phases in laser‐generated NPs. For instance, ablation with 800 nm (200 ps) pulses of In target in water originated a mixture of In(OH)_3_ and In_2_O_3_ NPs,^201^ although only In_2_O_3_ was observed by laser ablation of In in water with 532 nm (5 ns) pulses.^202^ The structural differences may be explained by the use of 200 ps instead of 5 ns pulses, because the use of ps pulses is associated with a shorter plasma lifetime and a colder plasma compared to ns pulses. Hydroxides are less stable than oxides at the high temperatures reached inside the plasma plume.^201^, ^202^ In fact, it has been suggested that hydroxide formation occurs in the last stage of LAL, or even after ageing in water of the NPs.^170^, ^201^ In general, the formation of hydroxides depends on the reactivity of the ablated material and solvent molecules in the specific synthetic conditions. For instance, brucite Zn(OH)_2_ platelets were obtained by LAL with 355 nm (10 ns) pulses of Zn in water,^203^ while ZnO is typically achieved with 532 and 1064 nm pulses.^141^, ^142^, ^143^ Gd(OH)_3_ was obtained by high energy 1064 nm pulses of a ns laser in aqueous environment.^204^ The relative yield of oxide versus hydroxide phases is influenced also by dissolved oxygen molecules in the liquid since LAL of Co in water or N_2_ purged water resulted, respectively, in CoO and β‐Co(OH)_2_ NPs, that spontaneously evolved into Co_3_O_4_ by ageing.^205^ Also, spindle‐like GaOOH particles slowly grew in a solution of CTAB during a few days after the ablation of a Ga target.^206^

Different types of surfactants can be used to promote or prevent the formation of metal hydroxides, since lamellar β‐Zn(OH)_2_ platelets were obtained in SDS (anionic surfactant) solutions,^203^ while ZnO NPs were observed in aqueous solutions of amphoteric (LDA), cationic (CTAB) and nonionic (OGM) surfactants (see Figure 7 A).^144^

Similarly, brucite Mg(OH)_2_ nanostructures were obtained by laser ablation with 1064 nm (5 ns) pulses of a Mg target in water or aqueous SDS solutions, while MgO NPs were found in organic solvents (acetone and 2‐propanol).^207^ Besides, the morphology of Mg(OH)_2_ nanostructures depended on the SDS concentration: ultrafine tubular‐like fibres were obtained at low concentrations of SDS solution, while stripe‐like rods and large platelets grew preferentially with increasing surfactant concentration.^207^

Ions are also important in determining the composition of the final products, as shown by LAL experiments at 355 nm (8 ns) of Cu or Zn targets in water and aqueous solutions of the corresponding metal salt (Cu^2+^ or Zn^2+^), but coupled with different counterions (CuCl_2_, Cu(NO_3_)_2_+NH_4_OH, ZnCl_2_+NH_4_OH, Zn(NO_3_)_2_). LAL in neat water generated mixtures of metal and metal oxide NPs, whereas nano‐paratacamite Cu_2_Cl(OH)_3_ was produced in aqueous CuCl_2_ solution, nano‐rouaite Cu_2_(NO_3_)(OH)_3_ was formed in aqueous Cu(NO_3_)_2_+NH_4_OH solution, nano‐simonkolleite Zn_5_(OH)_8_Cl_2_ **⋅**H_2_O formed in ZnCl_2_+NH_4_OH solution, and layered zinc hydroxide nitrate Zn_5_(OH)_8_(NO_3_)_2_ **⋅**2 H_2_O NPs formed in Zn(NO_3_)_2_ solution.^208^

Overall, it should be noted that a general problem encountered in the LASiS of oxide nanostructures may be the presence of non‐crystalline by‐products due to hydroxides or pyrolyzed solvent residuals, and the coexistence of multiple phases. Although amorphous phases can be detected by TEM, X‐ray diffraction or spectroscopic (FTIR, Raman, optical) analysis, the evaluation of the extent of non‐crystalline versus crystalline fractions is generally not possible or inaccurate by these techniques. LAL of Cu in water is such an example, since a mixture of CuO and Cu_2_O NPs has been frequently observed, and with a proportion changing with ageing time in favour of the more oxidized Cu phase.^209^ Another paradigmatic case is the LAL of Fe, since non‐crystalline iron hydroxides were evidenced by LAL in water, and there is a variety of iron oxides (wustite, maghemite, magnetite, hematite), which were observed in the same samples, even if a specific oxide phase (Fe_3_O_4_) was dominant.^118^, ^119^ Besides, amorphous carbon by‐products were found after LAL of Fe in various organic solvents, as evidenced by transmission electron microscopy and Raman analysis.^118^, ^119^, ^157^, ^158^, ^159^ This phenomenon was exploited for the generation of graphitic carbon‐encapsulated MnO, ZnO or Fe_3_O_4_ NPs by LAL in acetone,^210^ graphitic carbon‐encapsulated LiNbO_3_ NPs in toluene as well as chloroform^211^ and carbon‐encapsulated TiO_2_ after performing LAL in gaseous, liquid and supercritical CO_2_.^179^

To avoid the problem of non‐crystalline hydroxides in laser‐generated iron oxides, a simple etching procedure has been proposed, that is based on treatment with Ethylenediaminetetraacetic acid (EDTA) or diluted HCl,^118^, ^119^ which are easily washable compounds and allow to preserve the purity of the crystalline product at the end of the procedure. In this case, the NPs are collected by centrifugation, because the additives compromise the colloidal stability of the system.

In general, oxide nanostructures show limited colloidal stability at high concentration (≈mg mL^−1^) or long ageing times (typically days), unless stabilizing molecules are added during the synthesis, or the pH of the liquid is tuned far from the isoelectric point of the material. The decrease in colloidal stability over time can be due also to chemical modifications such as the reaction with atmospheric oxygen or the release of ions. The collection of laser‐generated oxides by centrifugation is easy in case of unstable colloids, and this electrostatic instability can be obtained by pH tuning towards the isoelectric point.

## LFL

### LFL fundamentals

In contrast to LAL, which requires bulk targets, LFL starting materials are micro‐ or nanoparticles dispersed in liquids. This prerequisite of LFL perfectly matches with the fact that basically all oxides are available in powder form. Generally, LFL has proven to be an effective method to gain small (∼10 nm) and ultra‐small (<3 nm) NPs, starting from an educt of larger NPs or microparticles dispersed in a liquid. LFL can be performed on ligand‐free educt particles,^114^, ^212^, ^213^, ^214^, ^215^, ^216^, ^217^, ^218^, ^219^, ^220^ or in the presence of stabilizing molecules,^152^, ^221^, ^222^ or other types of supports such as oxides.^152^, ^223^, ^224^, ^225^, ^226^, ^227^, ^228^, ^229^, ^230^, ^231^ Hence, the technique is generally applicable to colloidal NPs regardless of the respective synthesis method used. However, it is worth noting that the use of LAL‐generated educt NPs allows performing LFL in a chemical‐free way, to obtain clean size‐selected products.^17^, ^119^ During LFL, laser pulses with duration ranging from fs to ns and wavelength from visible to ultraviolet are applied to the liquid solution at laser fluences high enough to match the photo‐fragmentation threshold of the „educt“ particles (typically in the 1–100 mJ cm^−2^ range).^10^ In fact, contrary to LAL being performed with near‐infrared pulses in the majority of cases, LFL requires a careful selection of the appropriate wavelength, fluence, and pulse duration based on the band gap and size of the starting oxide particles, while avoiding a solvent breakdown.

The majority of LFL experiments are performed with a batch setup^10^, ^114^, ^152^, ^212^, ^213^, ^218^, ^219^, ^220^, ^221^, ^222^, ^225^, ^226^, ^227^, ^228^, ^229^, ^230^, ^231^, ^232^ employing a dispersion of the precursor NPs in a static, constantly stirred, or a fluxed cell with the laser beam being either unfocused or focused inside the cell. In the latter case, the focal plane is usually behind the cell to avoid liquid breakdown or entrance window damage.

By controlling the duration of laser irradiation, the number of pulses applied to each particle statistically depends on how often the NPs pass through the laser beam. Consequently, the average energy and number of laser pulses applied to the educt particles, as well as the related standard deviation, change if the liquid is stirred, fluxed, or is static and subjected only to convective mixing (non‐optimal condition). Indeed, only in a few cases in the literature,^214^, ^215^, ^216^, ^217^, ^223^, ^224^ the liquid is fluxed by a pump throughout the cell or a transparent tube, opening the question on how much the gradient with respect to the number of laser pulses applied to each nanoparticle affected the reports appeared so far.

**Figure 9:**
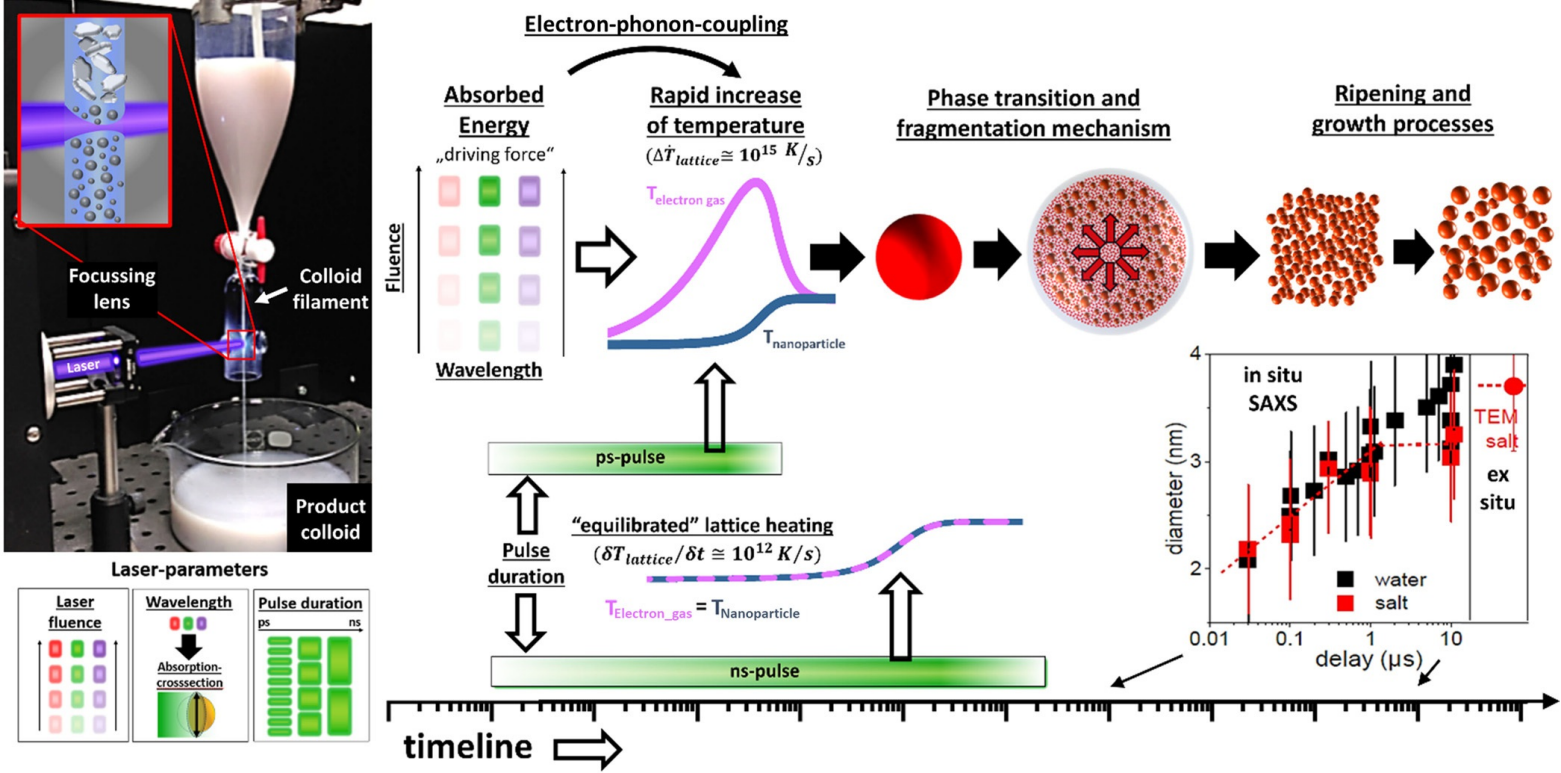
Left: A representative LFL set‐up in a free liquid jet (adapted with permission from ref. 37, Copyright 2018, American Chemical Society). Right: Conceptional overview of the main laser parameters and their relation to heating, phase transition, and ripening processes and relative timescale during LFL. The inset (bottom right) shows the growth process of a gold colloid after LFL, obtained by in situ SAXS (reprinted with permission from ref. 233, Copyright 2020, Royal Society of Chemistry).

To ensure that all particles are irradiated with a similar number of laser pulses, Lau et al. performed LFL in a free liquid jet (Figure 9).^223^ The jet is obtained from a capillary located at the bottom of a liquid reservoir containing the dispersion of educt particles, and the pulsed laser beam with defined laser fluences is applied at 90° to the liquid jet direction. In this way, the number of laser pulses applied to each volume of liquid passing through the capillary can be controlled by tuning the particle's residence time (inversely proportional to the applied flow rate through the illuminated part) and repetition rate of the laser.^37^, ^214^, ^223^ Finally, to account for fragmentation efficiency, the authors introduced the term „mass‐specific energy input“, describing the total amount of energy applied to the educt particle mass.^223^ This mass‐specific energy input is directly controlled by the number of laser pulses per liquid volume element. At given particle mass‐concentration this gives the number of laser pulses per educt‐particle. The latter can be controlled by the flow rate (or residence time of the educt‐particle inside the irradiated volume element) or the repetition rate of the laser. When using the passage reactor setup shown in Figure 9 constant number of laser pulses is applied to each nanoparticle with each passage through the irradiated volume element. With each passage, the number of laser pulses and hence mass‐specific energy input increases by the same value and, hence, it is precisely controlled.^214^, ^215^, ^223^ This approach allows a direct (mostly linear) tunability of particle size,^214^, ^223^ and particle properties (e.g., optical properties^224^ and band gap,^37^, ^223^ surface chemistry,^214^, ^234^ crystal phase^37^, ^234^ or catalytic activity^37^, ^224^, ^234^).

The basic steps leading to LFL are the following (Figure 9): light absorption by the initial NPs, heat transfer to the surrounding liquid (with plasma formation at highest irradiation fluence), phase transition and fragmentation, particle nucleation, growth and ripening. The individual fragmentation processes and steps (Figure 9) generally occur on an ultra‐short time scale ranging from ps (for heat transfer processes inside the solid) to ns and μs (for particle growth), although subsequent secondary ripening processes may last from seconds to hours and days.^235^ Hence, coalescence and re‐growth until reaching the thermodynamic equilibrium at ambient conditions are additional processes that need to be taken into account.^21^, ^212^ By adding ions (salts)^215^ or organic steric ligands^222^, ^236^ that coordinate the photo‐fragmented nanocrystals, regrowth can be limited in situ, resulting in smaller sizes compared to the ligand‐free particles.^236^ So far, the majority of mechanistic studies were focused on LFL of gold NPs as model material,^212^, ^213^, ^214^, ^215^, ^216^, ^218^, ^232^, ^237^ but LFL was employed for a broad range of particles (metals,^219^, ^220^ oxides,^152^, ^223^, ^225^, ^226^, ^227^, ^228^, ^229^, ^230^ nitrides,^227^ iodides,^231^ sulfides,^238^ semiconductors,^152^, ^223^, ^225^, ^226^, ^228^, ^229^, ^230^ organic crystals,^239^ hybrid perovskites^226^, ^236^). Fragmentation mechanisms are still under debate in literature but beyond the scope of this review. The consensus is that ns‐lasers mainly initiate photothermal evaporation processes and melting while ultra‐short laser pulses (≪10 ps) mainly lead to electron dynamic‐mediated mechanisms.^212^, ^213^, ^223^, ^232^, ^240^, ^241^, ^242^, ^243^ Most earlier mechanistic studies focused on post‐mortem analysis where only the final particle size after LFL (after ripening processes occurred) was investigated.^212^, ^213^, ^223^, ^232^, ^235^, ^240^, ^241^, ^242^, ^243^ New studies start to refine recent fragmentation mechanisms. Concerning the employed laser parameters shown in Figure 9, in situ methods like ultrashort X‐ray and electron diffraction^233^ allow to directly observe the fragmentation mechanism with high temporal resolution. Additionally, numerical studies provide more and more insight into the fragmentation mechanisms when using different laser parameters. For the case of fs‐400 nm‐laser pulses, Delfour and Itina found that metal NPs of ≈30 nm have the lowest fluence threshold for fragmentation.^244^ Similar size dependence of the threshold fluence was observed by Ziefuß et al. for ns‐532 nm laser pulses but discussed in terms of photothermal mechanism.^214^ Here, quantitative (i.e., nearly 100 %) fragmentation of Au NP was observed for educt particle size >40 nm. Experiments were conducted with single‐pulse per particle conditions.^214^ Interestingly, above the size threshold of ≈40 nm for educt particles, the quantitative formation of fragments with peak size located at 3 nm regardless if ns‐ or ps‐laser pulses were used. In the case of smaller educt particles (<40 nm) or lower laser intensities (<1.6 10^12^ W m^−2^ @ 53 nm educt‐particle size) a mixture of ≈3 nm and ≈13–20 nm NPs were observed. Formation of similar product particle size was also found for ns‐532 nm‐LFL of CoFe_2_O_4_ and BiFeO_3_ indicating a similar fragmentation mechanism.^224^, ^245^ Although LFL has been studied extensively in recent years, the effective nanoparticle productivity gained by LFL has barely been addressed in literature so far.

**Figure 10:**
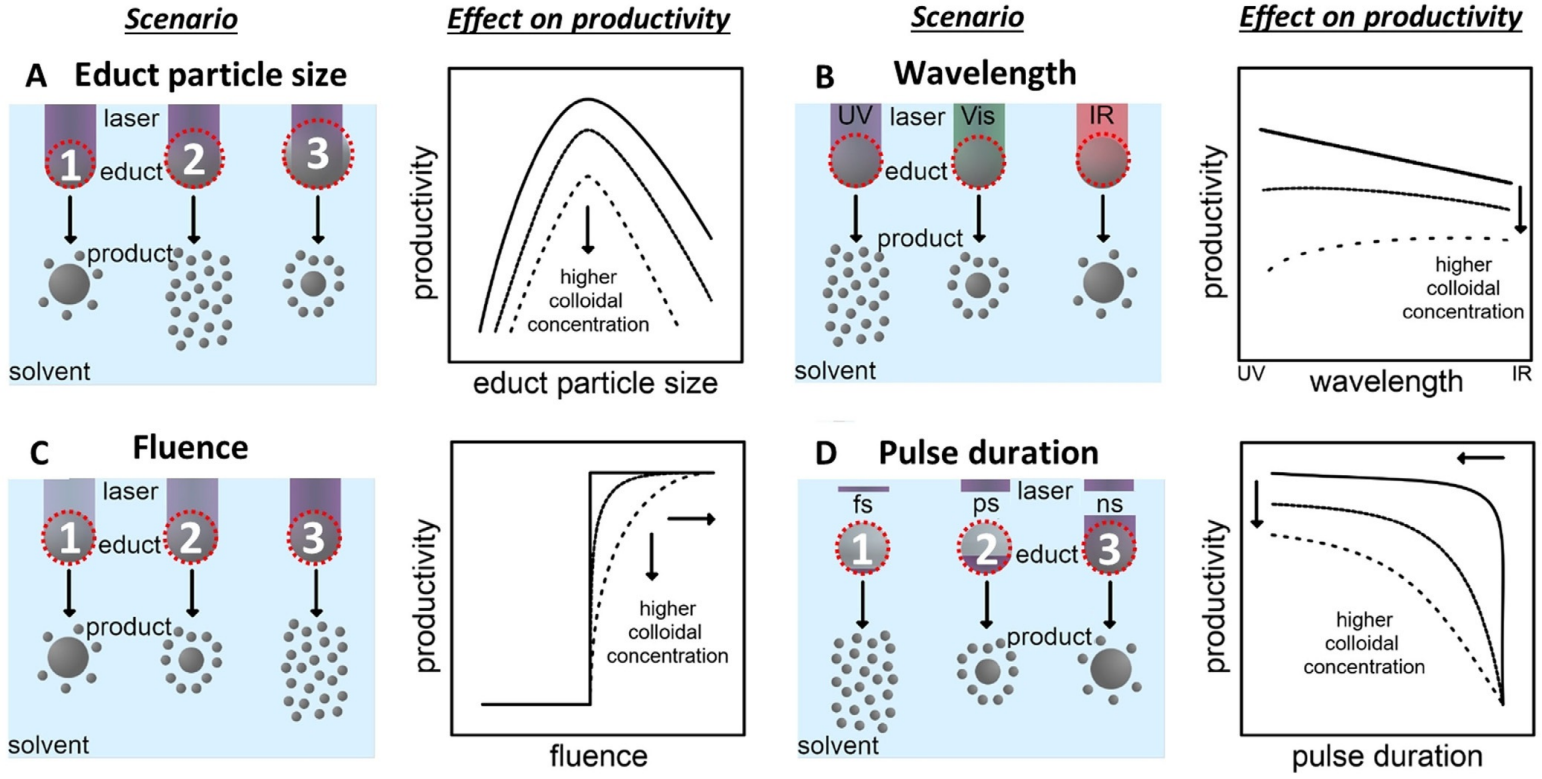
Illustrations on the effect of key laser parameters on nanoparticle productivity during LFL. The left column of pictures illustrated the scenario where the laser parameter is varied and the right column summarizes the general trend of productivity with the given laser parameters which are: A: educt particle sizes; B: wavelength; C: laser fluence; D: pulse duration. Adapted with permission from ref. 80.

In general, the main parameters influencing LFL may be summarized as shown in Figure 10:

A) the educt particle size, with an optimal range of 30–60 nm^214^, ^244^, ^246^, ^247^ but well above 10 nm,^214^ because the Rayleigh scattering of incident light by the colloid scales with the 6^th^ power of the particle diameter, while the absorption coefficient scales with the 3^rd^ power of the size;^248^

B) laser wavelength, because it affects the absorption and is usually larger at shorter wavelength;

C) the laser fluence, that is the driving force of the fragmentation process;

D) laser pulse duration, which sets the timescale of energy absorption versus heat loss dynamics.^80^

High productivity is possible with low nanoparticle concentration (Figure 10 A–D), intermediate educt particle size (Figure 10 A), short‐wavelength (Figure 10 B), high laser fluence (Figure 10 C) and short pulse duration (Figure 10 D). All this was combined in the work of Ziefuß et al. with single‐pulse‐per‐particle conditions.^214^ In addition to the determinants A)–D), the repetition is an important factor, even at constant laser power. The laser pulsing rate should be high enough to reach all particles within the irradiated volume, so that higher colloidal quality (narrower size distributions) is achieved using higher repetition rates, with the mechanism in a batch chamber being related to convective‐diffusion phenomena from and into the irradiated volume.^249^ In liquid flow, the repetition rate should be matched to the volume exchange rate within the intersection of laser beam and liquid flow channel, so that one volume element is hit at least once by a laser pulse. On the other hand, there must be an upper repetition rate limit, as the fragmentation event needs to at least proceed so far that the mother particles have enough time to be fragmented. Also, the cavitation bubble emerging during LFL should be temporally bypassed. Recently, temporally resolved, X‐ray‐based structural kinetic studies during LFL pointed at a LFL‐cavitation bubble lifetime of 50 to 100 ns, and the fragmentation product still growing after this period, on a hundreds of nanoseconds (to a few microseconds) scale.^233^ Accordingly, if a subsequent pulse is intended to be applied only after the fragmentation process caused by the previous pulse is terminated, the upper repetition rate limit of efficient LFL could be expected in the (few) MHz regime.

With the synthesis parameters given in ref. 214 (yielding ≈2 mg h^−1^ W^−1^) it can be estimated that a laser power of about 500 W is required to achieve productivities of ≈1 g h^−1^, that are enough for use in several „real world“ technological applications.^42^ Interestingly, this power regime appears to be independent of the pulse duration used. In the case of LAL, Streubel et al. achieved about 4 times higher productivity with the same laser power.^33^ Yet, for LAL, the NPs bear a much broader particle size distribution and potentially require post‐treatment,^43^ while NP after LFL are <3 nm in size.^42^ While this is only a first rough estimation, a critical comparison of the economic performance of LAL versus LFL needs to be conducted in future studies.

### Overview of oxide NPs obtained by LFL

**Figure 11:**
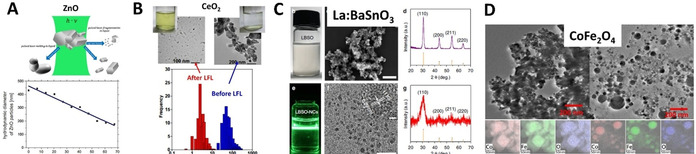
(A) LFL of ZnO in a liquid jet, showing the linear change of particle size with mass‐specific energy input (reprinted with permission from ref. 223, Copyright 2015, Elsevier). (B) Formation of 3 nm CeO_2_ particles by LFL of LAL‐generated submicron‐sized cerium oxide particles (reprinted with permission from ref. 152, Copyright 2014, Elsevier). (C) La:BaSnO_3_ particles with size efficiently reduced down to <10 nm while retaining the phase pure perovskite crystal structure (reprinted with permission under CC BY 4.0 from ref. 226, Copyright 2019, Springer Nature Ltd.). (D) CoFe_2_O_4_ NPs photo‐fragmented using ps laser pulses in the visible (reprinted with permission under CC BY 4.0 from ref. 224, Copyright 2017, Springer Nature Ltd.).

As previously discussed, LFL is an effective approach for size reduction and concomitant refinement of the size distribution of educt particles. Although the majority of publications on LFL (especially mechanistically ones) focus on noble metal NPs (mainly AuNP),^10^, ^212^ LFL of oxide‐based NPs has especially been used in application‐oriented publications. Several studies in literature showed that LFL is a powerful method for the introduction of defects in nanocrystalline oxides, that have found relevant applications in catalysis. For instance, 3 nm CeO_2_ particles obtained by LFL showed a significantly increased Ce^3+^ content of ≈40 % compared to the initial value of 7.5 %.^152^ The increased defect content was discussed in terms of a higher density of edges due to the small particle size after LFL, favouring the formation of stable Ce^3+^ defects of potential interest for catalytic studies.^250^ Lau et al. performed the LFL of ZnO in a liquid jet, showing the linear change of particle size and optical properties with mass‐specific energy input (Figure 11 A).^223^, ^239^ This was attributed to a shock‐wave mediated disaggregation and partial vaporization of the particles, which resulted to be several orders of magnitudes more effective than performing an ultrasonic treatment.^223^ Employing LFL of submicron‐sized cerium oxide particles using ns 1064 nm laser pulses with high energy (350 mJ), Takaeda et al. observed the formation of 3 nm CeO_2_ particles (Figure 11 B) that obtained high‐density oxygen vacancies and hence Ce^3+^ defects.^152^ Wang et al. performed LFL of La:BaSnO_3_ perovskite NPs with an initial size of 40 nm using ns 355 nm laser pulses at an intermediate laser fluence of ≈400 mJ cm^−2^.^226^ The authors observed an efficient reduction of the particle size down to <10 nm while retaining the phase pure La:BaSnO_3_ perovskite crystal structure (Figure 11 C). The fragmented NPs were subsequently embedded as co‐catalysts into Mo‐doped BiVO_4_ at gradually changing mass‐loadings, to achieve highly active photo‐anodes.^226^ The usage of La:BaSnO_3_ after LFL allowed to improve the photocurrent density by about 50 % without using hole scavenger, up to a level that is more than 80 % of the theoretically possible value.^226^ Consequently, oxide NPs after LFL do not only suit as ideal catalysts themselves, but also as co‐catalyst and components for the synthesis of supported catalysts. Though the choice of the appropriate precursor NPs is crucial, as shown by Schaumberg et al. at the example of laser‐based copper nanoparticle synthesis.^227^ Through LFL of CuO in acetyl acetate, the authors reported the formation of Cu/Cu_2_O core–shell NPs, while pure Cu NP formed on LFL of Cu_3_N NPs in the same liquid.^227^ Since the authors observed a carbon shell covering the NPs in the case of Cu_3_N, the occurrence of redox reactions during LFL appears to follow similar correlations as in the case of LAL,^180^ in agreement with other recent reports about metal alloys.^121^

## LDL

While LFL reduces the particle size, especially fundamental catalytic studies require a constant particle size to study structure–activity correlations. Hence, we propose the term Laser Defect‐engineering in Liquid (LDL, Figure 1 D) to summarize all studies that aim to alter materials properties and defect densities without any significant change in particle size. In LDL, defects must be introduced in the final product, that are not present in the pristine particles. Therefore, there must be some experimental evidence about the change in defect density as the main effect of the laser treatment, to verify the occurrence of LDL. When considering the laser‐induced size‐tailoring methods (LFL, LML), one can identify three different situations:

1) structural changes do not occur during laser‐induced size tailoring;

2) structural changes occur during size tailoring as the effect of the laser treatment but are connected to phase or morphological modifications, not to defect engineering;

3) defect engineering occurs simultaneously to size‐tailoring, thus the process falls in an intermediate case between LFL/LML and LDL.

Zuniga‐Ibarra et al. performed LDL of TiO_2_ NPs using high intensity 532 nm, ns‐laser pulses observing the transformation of anatase to rutile accompanied by a reduction of band gap from 3.2 eV down to 1.8 eV and the formation of amorphous TiO_2_, likely located near the surface of TiO_2_ particles,^228^ similar to other studies using UV‐laser pulses.^37^, ^229^ So far the formation of black TiO_2_ has only been reported under hydrogen treatment and explained by hydrogen‐induced n‐doping of the semiconductor, leading to significantly increased conductivity, defect‐related tailoring in the conduction and valence band,^228^, ^251^ as well as broadband absorption and improved photocatalytic activity.^228^, ^250^ Since the educt TiO_2_ NPs were polydisperse, Zuniga‐Ibarra et al. observed simultaneous LFL of larger particles and LML of smaller ones. This highlights the importance of using educt particles with narrow size distribution and avoid laser fluence gradients for effective LDL. The negative effect of such fluence gradients was indicated by Waag et al., who assigned occurring phase segregation processes to the former after performing laser beam ray‐tracing calculations.^224^ Further, it has been highlighted before, that using a cylindrical lens in case of the liquid jet setup is helpful to achieve better illumination, as demonstrated for the laser‐based tuning of ITO optical absorbance.^252^ Consequently, shaping the naturally circular liquid jet cross‐section into a more flat geometry using an elliptical nozzle has recently been reported to improve the energy input during laser irradiation of oxide particles.^245^

A further study focused on the effect of laser treatment of TiO_2_ and ZnO NPs to tune their defect density and materials properties. As final readout, the exhibited photocurrents were measured for samples treated with a different number of ns‐UV‐ or ps‐VIS‐laser pulses per particle.^37^ Interestingly, for ns‐UV‐laser pulses the authors observed a decreasing photocurrent in all cases. Here the smallest number of pulses that were used in the study was ≈70 pulses per particle. From an extensive analysis, the formation of bulk defects due to thermally initiated isochoric melting (typical of ns‐pulses) was proposed for all cases. When the same educt material was treated with a small number of ps‐VIS‐pulses (<150 pulses per particle), the performances/photocurrents improved by a factor of two. This was attributed to preferential absorption of the laser pulse at pre‐existing defects during LDL (since, wavelength: 532 nm→2.3 eV<*E*_BG_(TiO_2_)=3.2 eV) improving exciton generation during measurement of photocurrents. For a larger number of applied ps‐VIS‐laser pulses (>150 pulses per particle), the photoluminescence and photocurrents decreased as it was observed in all experiments using the ns‐UV‐laser. Here, segregation and movement of the defects to the surface were discussed as a reason for the decreasing photocurrents at a higher number of laser pulses.^37^

Note that, in the above studies, the change of colour in TiO_2_ has always been accompanied by rutile formation and has only been observed when ns‐laser pulses have been used, regardless of its wavelength.^37^, ^228^, ^229^ For this reason, isochoric melting altering crystal structure and colour of TiO_2_ was discussed for ns‐laser pulses.^37^ Yet, irradiation with ps‐laser pulses also led to enhanced photocatalytic properties, though without any colour change.^37^ Therefore, the fundamental processes behind defect formation still deserve further investigation.

As previously indicated by the LFL experiments of CuO in acetylacetate, the high temperature reached by the educt NPs also promotes solvent degradation and redox reactions. Depending on the nature of the involved chemical substances, the reduction or oxidization may be favoured.^19^ This process can be seen as the nanoscale version of what happens microscopically in the ablation plume during LAL. The origin of such redox reactions in LFL has been discussed in the literature,^253^ but it has not yet been addressed in a systematic study, especially not in terms of LDL. Since LDL aims to maintain the particle size, defect formation can only be introduced by photo‐induced redox reactions. Understanding and controlling these is hence of utmost importance for efficient LDL. A first indication of the underlying mechanisms can be drawn from the recent studies of Waag et al. The authors fragmented CoFe_2_O_4_ NPs using ps 532 nm laser pulses and the liquid jet setup (Figure 9).^224^ The particle size of CoFe_2_O_4_ was nearly constant (slightly reduced) but with clear morphology changes from rather cubic to spherical shape after LFL. The homogenous elemental distribution has been maintained (Figure 11 D). Yet, the optical properties were tuned and electrocatalytic activity significantly enhanced. That was attributed to the formation of CoO and layered Co di‐hydride during LFL, the latter being associated with the sheet‐like background observed in TEM analysis of the samples after LFL.^224^ Hence, reduction processes must have taken place. They discussed that the decomposition may have been induced by self‐focusing of the laser when being refracted at the liquid jet.^224^ The authors recently performed LFL of BiFeO_3_ in water and propylene carbonate using an elliptical liquid jet (instead of a spherical one) to improve the homogeneity of irradiation. The authors successfully reduced the particle size from initially 450 nm down to 10 nm but still observed partial decomposition of the BiFeO_3_ forming additional iron oxides regardless of the solvent used.^245^ Hence, the origin of phase decomposition remains still an open question.

Although oxidation is more frequently observed in literature during laser treatment of metal NPs in water, two other independent studies of LFL of Co_3_O_4_ also observed the reduction of Co‐ions from Co^3+^ to Co^2+^ accompanied by the formation of oxygen vacancies^254^ and CoO,^234^ Hence, one may conclude that laser processing of oxide NPs is a reductive process. In the study of the Tüysüz group,^234^ the authors also studied CoO particles apart from the Co_3_O_4_ spinel mentioned before. Interestingly, after the laser processing of CoO, a fraction of Co_3_O_4_ spinel was observed in the products, now indicating oxidation of Co^2+^ ions to Co^3+^. Consequently, for the same element the occurrence of oxidation or reduction during LDL in water appears to depend on the initial state of oxidization of the educt particles. Note that the educt material of the Tüysüz group is derived from templating chemistry of coffee waste, connecting laser synthesis even more tightly with green chemistry and circular economy.

The interplay of initial oxidization state, redox potentials and laser parameters are still unclear. However, seminal laser studies about laser irradiation of colloids appeared, where the laser fluence is chosen to be low enough to maintain the initial oxide particle size but high enough to induce redox reaction between liquid media and NPs.^19^, ^37^ In these studies, the band gap, photoluminescence, and photocatalytic activity of TiO_2_ and ZnO NPs were laser‐modified independent of the particle size, due to the alteration of location and density of defects.^19^, ^37^ It is expected that LDL will not only significantly impact studies of structure/defect‐activity independent of particle size but also be an efficient tool to study fundamental redox‐based processes occurring during laser excitation of oxides.

## LML

### Mechanism and processing conditions

Pulsed laser melting in liquid is a technique in which laser irradiation of a starting particulate material dispersed in a liquid is utilized to induce a thermal process, particularly melting, to fabricate new particles. This melting can be either isochoric (through re‐shaping particles like rods, polygons or fusion or pre‐existing aggregates) or cause size increase (through fusion of aggregates forming during LML) referred to the starting particle diameter. LML is also known as laser‐induced reshaping, and it differs from the LAL process because the fluence used for LML is one to two orders of magnitude lower than that of LAL. Suspended particles are generally used as raw materials for LML, so the laser beam is directed (usually unfocused and occasionally focused depending on the fluence required) onto a liquid dispersion of the raw particles. The shape of the particles is modified by the laser‐induced thermal process. Thus, plasma emission and the generation of shockwaves commonly observed in LAL are not evident during LML.

**Figure 12:**
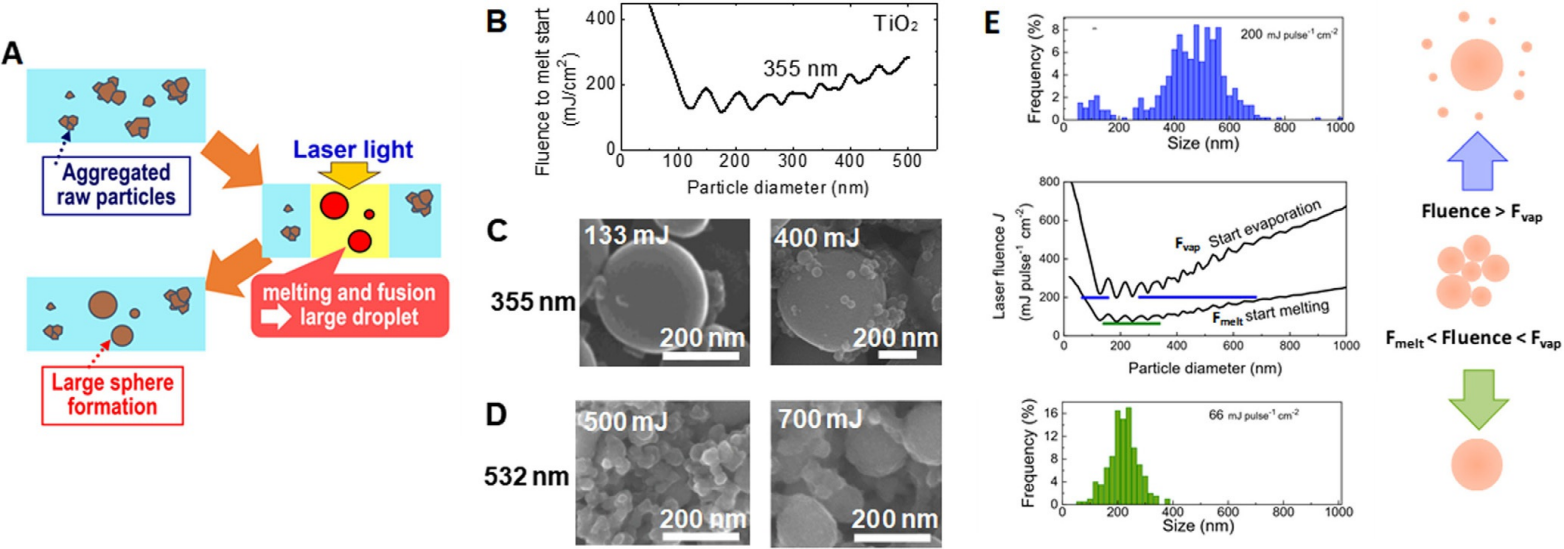
(A) Schematic illustration of the basic LML process. (B) Size‐fluence diagrams for TiO_2_ under irradiation with a ns 355 nm laser. SEM images of TiO_2_ particles obtained (C) with a 355 nm laser at a fluence of 133 and 400 mJ pulse^−1^ cm^−2^, and (D) with a 532 nm laser at a fluence of 500 and 700 mJ pulse^−1^ cm^−2^. (E) Size distributions of spherical TiO_2_ particles irradiated with a 355 nm laser at 200 mJ pulse^−1^ cm^−2^, and 66 mJ pulse^−1^ cm^−2^. Size‐fluence diagram of TiO_2_ under irradiation by a 355 nm laser after correcting particle absorbance changes by laser irradiation. Particle size ranges of the experimentally obtained data are shown in blue crossbars for 200 mJ pulse^−1^ cm^−2^, and a green crossbar for 66 mJ pulse^−1^ cm^−2^. Corresponding schematics are illustrated on the right side.

A schematic illustration of the basic LML process is shown in Figure 12 A. When particles absorb light energy from the pulsed laser, only the temperature of the particles increases rapidly. This is due to the transparency of the surrounding liquid and the small heat capacity of the raw particles. If the temperature of the particles exceeds their melting point, they form transient molten droplets. Then, in the interval between different pulses, they are quenched by the surrounding liquid medium and form solid spherical particles.

The formation of sub‐micrometre spherical oxide particles was initially reported for TiO_2_,^255^ although with very low yield. In subsequent studies, similar submicrometric particles were occasionally found in LAL products as minor unwanted by‐products.^256^, ^257^ Their origin has not yet been uniquely identified, being possibly formed by irradiation of particles agglomerates or large target fragments at the laser beam edge, usually with a Gaussian profile, which might be in an appropriate fluence range for LML. They might also come from the ejection of melted target droplets during target ablation (i.e., at high effective laser fluences during LAL^22^). The term sub‐micrometre particles refers to hundreds of nanometre particle diameter, which is a typical size range obtained by LML. The intentional formation of sub‐micrometre spherical particles as main products, by the LML technique, was reported only a decade ago for carbides,^258^ and metals,^17^ and later achieved with various oxide materials,^252^, ^259^, ^260^, ^261^, ^262^, ^263^, ^264^ even in the size range of tens of nm,^119^ or for the incorporation of Au NPs into ZnO starting from AuNP supported on ZnO.^262^ The terms “sub‐micrometre particle” or sub‐micrometre sphere (SMS) refer to hundreds of nanometre particle diameter, which is a typical size range obtained by LML, where in most cases perfectly spherical and (poly)crystalline particles are produced.

The pulsed lasers required for LML have pulse widths on the order of nanoseconds or picoseconds, and their fluences range from 10 to 200 mJ pulse^−1^ cm^−2^ depending on the material. Due to heat dissipation, a higher fluence is needed to fabricate sub‐micrometre spherical particles using long‐pulse lasers with pulse widths of several tens of nanoseconds.^265^ If picosecond pulses with the same wavelength and energy are used for LML of low‐thermal‐conductivity ceramic materials, the obtained sub‐micrometre spherical particles will be smaller than with nanosecond pulses.^266^ The reason is that, with ps pulses, heating occurs in a particle on a shorter time scale than heat dissipation to the surrounding, which usually requires ns. Thus, ps pulses are associated with a higher heating efficiency of pristine agglomerates of NPs, such that the melting temperature is reached at a smaller size compared to ns pulse irradiation with the same wavelength and energy.

Another laser parameter relevant for the final particle size is the laser fluence.^259^ No obvious morphological changes are observed at a lower laser fluence, while higher laser fluence will induce the formation of NPs from the raw particles through vaporization, setting intermediate conditions between LML and LFL. This stepwise size change from raw particle size to the sub‐micrometre scale, then down to nanometres with the laser fluence increase, well corresponded to the phase change from a solid to a liquid and finally to a vapor by particle temperature increase. Therefore, appropriate fluence control is necessary to selectively obtain sub‐micrometre spherical particles and stay in the LML range without falling in intermediate LML/LFL operating conditions.

The fluence threshold to fabricate sub‐micrometre spherical particles via LML process is approximately calculated based on the optical and thermal properties of the materials and the assumption of an adiabatic process.^16^, ^267^ That is, the laser energy supplied to the particles is exclusively used to heat the particles from room temperature to their melting point without dissipation of heat to the surroundings. The laser fluence, (mJ cm^−2^), required to start the melting of a single spherical particle can be calculated using Equation (2):

(2) $$J\left(d\right)=6.67x10^{-2}\rho_{p}\Delta \tilde{H}\frac{d}{Q_{abs}^{\lambda }\left(d\right)}$$

where *ρ*_p_(g cm^−3^) is the particle density, Δ*H̃*(kJ g^−1^) is the enthalpy from room temperature to the melting point, *d* (nm) is the diameter of the particle, and $Q_{abs}^{\lambda }\left(d\right)$ is the absorption efficiency (absorption cross‐section divided by geometrical cross‐section) of the particle‐based on the Mie theory. A size‐fluence diagram can be then obtained to visualize the relationship between the laser fluence (*J*) used to fabricate the spherical particles and the resultant particle size (*d*).^267^

Size‐fluence diagrams for the start of TiO_2_ melting with the commonly used 355 nm (wavelength) Nd‐YAG lasers are shown in Figure 12 B. The 355 nm laser can melt TiO_2_ particles and yield sub‐micrometre spherical particles at a lower fluence than in the visible range. This is due to the relatively higher $Q_{abs}^{\lambda }\left(d\right)$ at 355 nm wavelength compared to the visible light range, which derives from the difference between the complex refractive indices of TiO_2_ and reflects the relationship between photon energy and the TiO_2_ band gap.^261^, ^268^ The gradual fluence increase with increasing particle size in Figure 12 B, that is apparent for sizes above 300 nm for 355 nm wavelength, is due to the nearly constant absorption efficiency $Q_{abs}^{\lambda }\left(d\right)$ in the large particle size range, where geometric optics becomes dominant. In contrast, a drastic increase of fluence curves for particles smaller than 100 nm is attributed to the steep decrease in $Q_{abs}^{\lambda }\left(d\right)$ when particle size is much smaller than the laser wavelength.

This trend is common among most insulating or semiconducting oxides, and the fluence minimum for melting with a 355 nm laser is usually found at diameters of 100 to 200 nm. Particles in this size range are thus easily melted by applying a fluence that exceeds the calculated minimum value, and the diameters of particles obtained via LML are often in this range. Scanning electron microscope (SEM) images of TiO_2_ particles obtained at different fluences by 355 nm and 532 nm laser irradiation are shown in Figure 12 C and D. As expected, the images indicate the formation of sub‐micrometre spherical particles at 355 nm in this size range and the increase of size with increasing fluence due to the repetitive melting and fusing. At 532 nm, due to the lower optical absorption, a fairly large fluence is required for the formation of spherical particles. Therefore, suitable laser wavelengths must be selected to fabricate sub‐micrometre spherical particles at lower laser fluences.

The size‐fluence diagram is also closely related to the obtained particle size distribution.^268^ The size distributions of sub‐micrometre spherical TiO_2_ particles obtained with a 355 nm laser at fluences of 200 and 66 mJ pulse^−1^ cm^−2^ are shown in Figure 12 E. Nearly monodisperse sub‐micrometre spherical particles were obtained at 66 mJ pulse^−1^ cm^−2^. In contrast, laser irradiation at 200 mJ pulse^−1^ cm^−2^ yielded quite large spherical particles (400–600 nm) along with small particles ≈100 nm in diameter. Particles with diameters of ≈200 nm were absent. Size‐fluence curves showing the start melting and start vaporization of TiO_2_ under irradiation with a 355 nm laser after correction for changes in absorbance by reduction of TiO_2_ during irradiation are shown in Figure 12 E. The start vaporization curve was obtained by substituting Δ*H̃* for the total enthalpy from room temperature to the boiling temperature including the latent heat of melting. The blue and green crossbars in Figure 12 E indicate the presence of spherical particles and nearly correspond to the melt‐phase formation range in the size‐fluence diagram. The absence of 200 nm particles after irradiation at 200 mJ pulse^−1^ cm^−2^ can be attributed to the size‐selective vaporization of particles into NPs. The relationship between the size of fabricated particles and laser fluence can thus be explained semi‐quantitively using size‐fluence curves, though this assumes an adiabatic process and the dependence of *ρ*_p_ and $Q_{abs}^{\lambda }\left(d\right)$ on temperature is not considered. Heat dissipation can be largely ignored in oxide particles smaller than 100 nm in diameter due to their low optical absorption. Therefore, the conditions for sub‐micrometre spherical oxide particle formation can be effectively estimated using the size‐fluence curves obtained with Equation (2).

The discussion so far has considered only photo absorption by single particles and the melting of individual particles under laser irradiation. However, laser irradiation in the LML process clearly increases the size of „educt“ particles from nanometres to the sub‐micrometre range. If the particles in the liquid medium are aggregated, some of the constituent particles are melted and merge to form larger particles under laser irradiation. Thus, the aggregation of raw particles in the dispersion medium has an important role in the formation of larger sub‐micrometre spherical particles.^269^, ^270^

#### Photo‐absorption‐assisted LML

**Figure 13:**
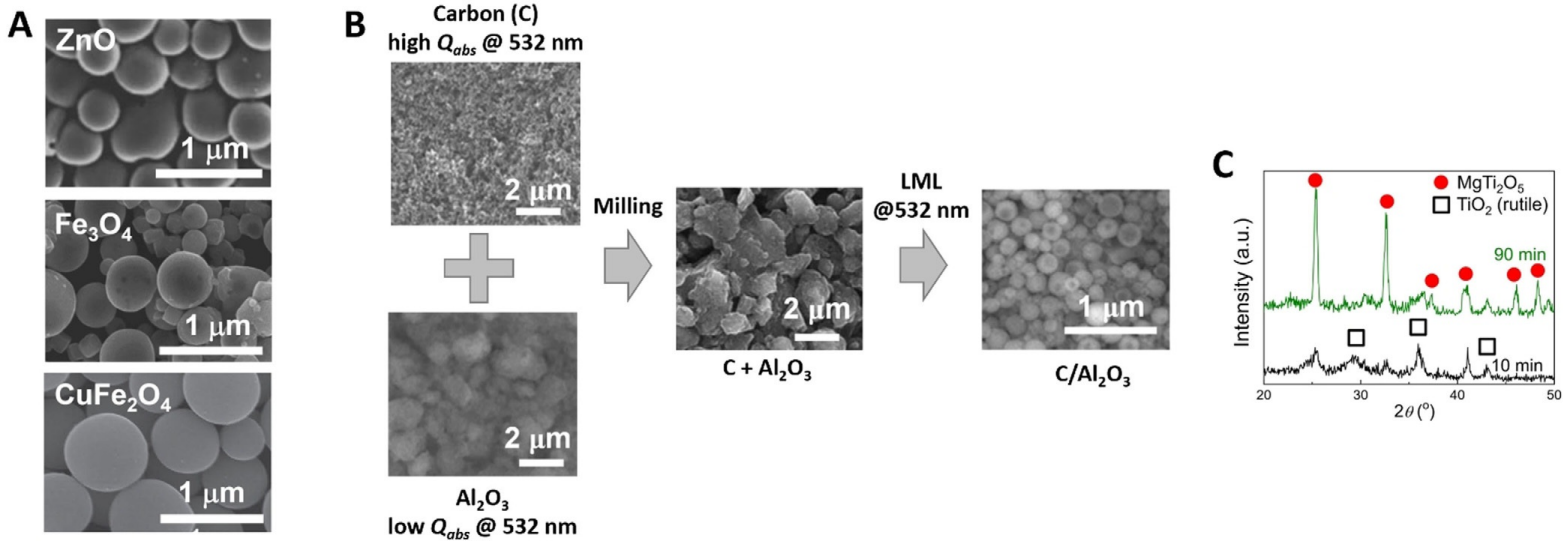
(A) SEM images of typical sub‐micrometre spherical oxide particles obtained via LML. (B) SEM images of raw carbon, raw Al_2_O_3,_ an Al_2_O_3_/carbon milled mixture before laser irradiation, and the mixture after laser irradiation (355 nm, 460 mJ pulse^−1^ cm^−2^, 30 min). (C) Irradiation time dependence of XRD patterns of particles obtained via LML of a milled mixture of 1:1 MgO and TiO_2_. Sub‐micrometre spherical MgTi_2_O_5_ particles were formed at 133 mJ pulse^−1^ cm^−2^ for 90 min by reactive LML fabrication of a complex oxide from single oxide constituents. Reprinted with permission from ref. 277.

Sub‐micrometre spherical oxide particles can thus be fabricated through LML using materials that are capable of sufficient optical absorption and that do not sublimate but melt. An appropriate selection of laser wavelength and fluence is important for sub‐micrometre spherical oxide particle fabrication. SEM images of typical oxide particles fabricated via LML are shown in Figure 13 A, including ZnO,^271^ Fe_3_O_4_,^272^ and the complex oxide CuFe_2_O_4_.^273^

As described above, enough optical absorption is required to fabricate sub‐micrometre spherical particles. However, various insulating oxides have band gaps larger than 5 eV, and in these cases, shorter laser wavelengths are required to ensure optical absorption at the level required for melting. Unfortunately, optical absorption in the liquid phase cannot be ignored at shorter wavelengths, often making impossible the selective heating of the raw particles. Conversely, if LML is attempted on materials with large band gaps using a 355 nm or 532 nm laser, the fluence required to melt the particles would exceed several J pulse^−1^ cm^−2^. The fluence would approach the typical ablation threshold, even if a laser with a nanosecond pulse width is used. Thus, a different approach is needed to enhance effective photoabsorption, consisting of LML assisted by close contact with photo absorber particles. SEM images that illustrate the fabrication of sub‐micrometre spherical Al_2_O_3_ particles with the assistance of photo‐absorptive carbon NPs are shown in Figure 13 B.^274^ Close contact between the photo absorption‐assisting carbon particles and the non‐photo‐absorptive raw particles is achieved by co‐milling.

This contact is needed to indirectly heat the raw material to obtain sub‐micrometre spherical particles by LML. This technique can be applied to other materials with large band gaps, like ZrO_2_,^274^, ^275^ MgO,^274^, ^275^ and yttria‐doped ZrO_2_.^276^

By extending this idea, sub‐micrometre spherical particles of complex oxide MgTi_2_O_5_ can be reactively fabricated, mimicking conventional ceramic processing.^277^ Photo‐absorptive TiO_2_ and less photo‐absorptive MgO were mixed and milled together to ensure close contact, and the mixed powder was irradiated with a 355 nm laser. The X‐ray diffraction (XRD) pattern of MgTi_2_O_5_ was observed in the pattern of the LML product (Figure 13 C).

#### Upscaling LML

Most LML experiments are performed by irradiating a colloidal suspension of raw particles in a batch cell with a pulsed laser while agitating the suspension with a magnetic stirrer or an ultrasonicator. Irradiation is typically performed for 5–30 min using a 10–30 Hz laser with an unfocused beam, and sub‐micrometre spherical particles can be obtained in over 90 % yield at production rates of several mg h^−1^. LML can also be performed by irradiating free‐fall liquid flow with a high‐repetition laser with a picosecond pulse width.^32^ This idea was recently extended to slit‐flow irradiation, and sub‐micrometre spherical particles were obtained in over 90 % yield after just one passage.^278^ The number of irradiating pulses can be precisely controlled in this process, and the flow system has been applied to analyse the initial stage of particle formation.^272^

## Applications of Laser‐Generated Oxide NPs

### Catalysis

#### Metal oxides in photocatalysis

Metal oxides (MOs) are widely employed as catalyst for oxidation catalysis,^19^, ^279^, ^280^ electrocatalytic,^224^, ^281^, ^282^, ^283^ and photocatalytic pollutant degradation^284^, ^285^, ^286^ in our living environment. As a prominent example, a variety of MOs (e.g., TiO_2_, WO_3_, NiO) in the pure and mixed forms have been utilized in the heterogeneous photocatalytic depollution of air and water. Light‐induced generation of electron/hole pairs, migration of the carrier at nanostructure surface, and the reactive events at the interfaces, are the key steps for a photocatalytic process. For this reason, the photocatalytic efficiency is generally dictated by the light‐absorption efficiency, which determines the number of photo‐generated charge carriers and their separation, while electron‐hole recombination is a competing process, decreasing the carrier mobility and preventing carriers from reaching the surface.

In this scenario, laser‐assisted synthesis of oxides in liquids can be considered a high‐quality strategy to generate clean oxide NPs, to stabilize these particles and to obtain heterogeneous catalysts thus improving the catalytic performances of colloidal samples prepared by classical chemical methods.

Generally, metal oxide films and nanostructures prepared by wet‐chemical processes require a multistep approach involving high temperature or high‐pressure conditions to achieve the optimal catalytic behaviour. These treatments address several features, among them the removal of solvents and binding agents, phase and microstructure changes, sintering and densification, the introduction of defects and oxygen vacancies. Regarding all photocatalytic processes, defects and band gap engineering is crucial for tailoring the specific features of the semiconductor oxide with the radiation which enhances the catalytic activity and the adsorption/desorption of reactants.^229^, ^250^, ^284^, ^285^, ^287^ In this respect, LAL has the potential to provide easy and green ways to achieve optimal results with minimal efforts. As an example, LAL‐generated ZnO NPs exhibited interstitial sites and oxygen vacancies (Frenkel/Schottky and ionization reactions),^288^ which were evidenced through green and blue photoluminescence effects.

The use of laser ablation was also exploited by Kohsakowski et al.^289^ for the preparation of TiO_2_‐CuO_*x*_ and TiO_2_‐FeO_*x*_ composites with photocatalytic rates in the degradation of 2,4‐dichlorophenoxyacetic acid enhanced by a factor of ≈1.5 under solar irradiation. The authors generated „naked“ (ligand‐free) NPs of CuO_*x*_ or FeO_*x*_ by LAL of metal oxide targets in water, followed by colloidal deposition of CuO_*x*_ and FeO_*x*_ NPs onto anatase TiO_2_, by adjusting the pH to establish electrostatic attraction between the colloids and the substrate.

**Figure 14:**
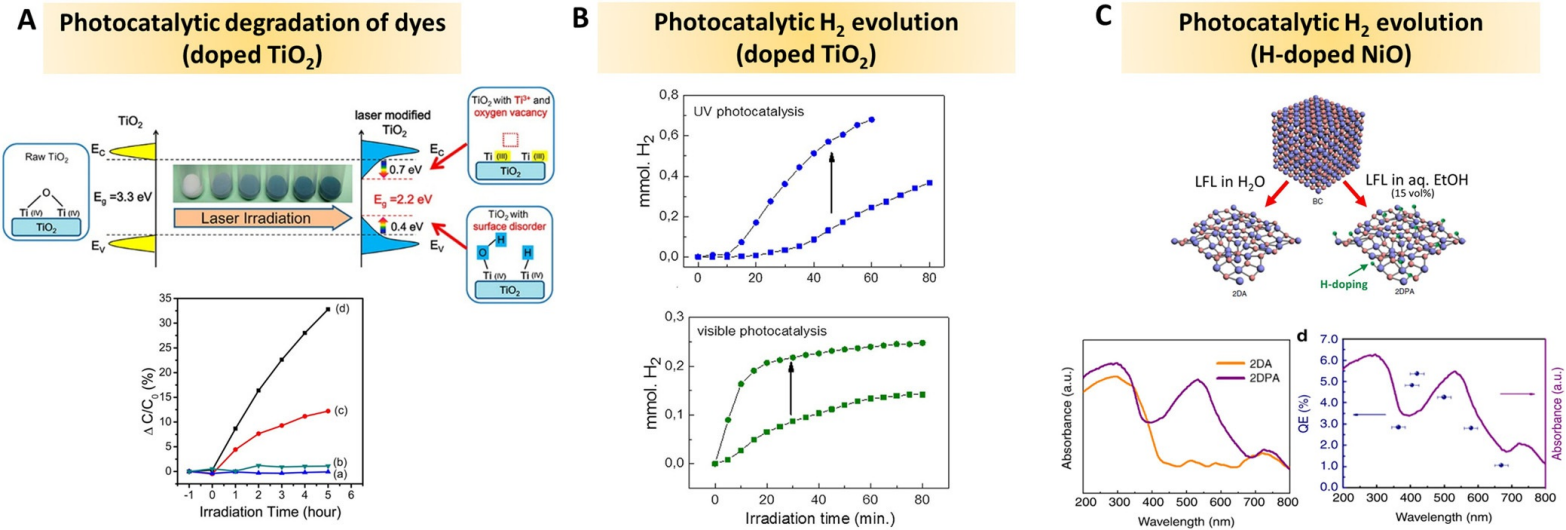
(A) Defective blue Titania containing surface defect states as under‐coordinated Ti^3+^ sites and oxygen vacancies. High photocatalytic activity towards the degradation of rhodamine B has been demonstrated for defective laser‐generated titania (lower panel, see text for description; reprinted with permission from ref. 229, Copyright 2015, American Chemical Society). (B) Hydrogen production under UV or visible irradiation of defective titania NPs (reprinted with permission from ref. 287, Copyright 2017, Elsevier). (C) LFL of NiO in pure water or an aqueous solution of ethanol originated amorphous 2D sheets that, in the latter case, exhibit intensive H doping and plasmonic properties useful for efficient light absorption (upper graph) and impressive quantum efficiency in H_2_ evolution by solar irradiation (lower panel); reprinted with permission under CC BY 4.0 from ref. 253, Copyright 2018, Springer Nature Ltd.).

Among the binary oxides (e.g., TiO_2_, ZnO, SnO_2_), TiO_2_ NPs are the most investigated photocatalysts due to their low cost, inert nature, and photostability.^285^ The overall photocatalytic activity of TiO_2_ is determined by its crystalline structure, surface area, density of surface hydroxyl groups and adsorption/desorption characteristics. Anyway, its main drawback is the fact that its efficiency under visible irradiation is low due to its large band gap, and it can absorb only the UV radiation, which is about the 5 % of solar radiation, thus its use on large scale is limited. Moreover, the fast recombination of photo‐produced electron‐hole pairs leads to low photo‐efficiency. Processing such as doping and self‐doping (reduction) through hydrogenation or addition of sacrificial agents can improve the catalytic performances, even though such classical approaches relying on the use of gaseous H_2_ are dangerous and difficult to control. For these reasons, TiO_2_ has been treated by lasers directly in the colloidal form.^37^ This produces defective blue titania,^37^, ^229^ which contains surface defect states as under‐coordinated Ti^3+^ sites (Figure 14 A) and oxygen vacancies which play a crucial role in the electron injection and recombination dynamics resulting in increased photocatalytic activity.^290^ Chen et al.^229^ have demonstrated that laser irradiated colloidal TiO_2_ NPs provide enhanced photocatalytic activity towards the degradation of rhodamine B, a model dye molecule for this kind of studies.^291^ The curves reported in Figure 14 A represent the photocatalytic degradation of rhodamine‐containing water solution under green irradiation (LED, centre wavelength 520 nm) for P25 (a mixture of rutile and anatase crystal phases, curve (a)), raw TiO_2_ (curve b), TiO_2_ laser‐treated for 60 min (curve c), and TiO_2_ laser‐treated for 120 min (curve d). Similarly Filice et al.^287^ have shown that the same treatment for titania increases the photocatalytic water splitting performances of TiO_2_ under UV action. As reported in Figure 14 B, the results pointed to an enhancement of up to a factor of three for hydrogen production under UV irradiation and twofold under visible irradiation, compared to the non‐irradiated titania, although the total amount of hydrogen production with visible light is too low for „real‐world“ applications. Also, catalyst durability (recyclability) studies are needed to validate its application potential. The same authors observed that the modifications induced by laser irradiation, that are responsible for the increased photocatalytic activity, depend on the laser process parameters. At a given irradiation wavelength and for nanosecond pulses, a laser fluence increase acts linearly by increasing the amount of hydrogen produced per unit time. Such a behaviour has been correlated to the parallel increase of under‐coordinated Ti ions which have consequences in the modification of the electronic structure.^287^ Moreover, fundamental roles of the liquid in which the catalyst is embedded have been recently evidenced by a series of experimental findings, supported by ab initio molecular dynamics simulations which involve water and ethanol. Ethanol molecules strongly passivate surface oxygen vacancies while water only weakly interacts with this surface. Then a correct balance between the two liquids should be properly considered either for the laser modification of the materials and for the catalytic experiments.^292^

LFL has also been used to produce materials suitable for plasmon‐enhanced photocatalysis, which is typically investigated by using plasmonic noble metal NPs loaded onto semiconductor oxides.^37^, ^293^, ^294^, ^295^ Besides, Lin et al. were able to form 2D nanosheets by LFL of NiO NPs in a methanol/water solution.^253^ LFL of NiO in pure water or an aqueous solution of ethanol always originated amorphous 2D sheets with plasmonic behaviour and superior photocatalytic activity and stability compared to the not irradiated material, boosting quantum efficiency of H_2_ evolution by solar irradiation (Figure 14 C).^253^ Indeed, the reducing environment created by ethanol promotes the formation of Ni^0^‐like defects with plasmonic properties by hydrogen n‐type doping of the nanosheets, thereby raising the Fermi level above the conduction band edge, thus leading to the appearance of surface plasmons at visible light frequencies.^253^ On the contrary, LFL in pure water led to a narrowing of band gap due to additional defect formation but was not able to overcome the intrinsic p‐doping of NiO.^253^ It is therefore evident how the choice of the liquid during LFL is of utmost importance, although it was less investigated than for LAL.^19^

#### Supported redox heterogeneous catalysts

Laser processes in liquids are particularly efficient to load transition metal NPs or clusters onto oxide NPs.^19^ Either the supporting species (oxide) and/or the metallic particle can be separately generated by LAL and then mixed under to obtain the final heterogeneous catalyst. Metal oxide NPs, as well as other forms of oxides such as graphene oxide, are by far the most prominent supports for (co‐)catalytic species (mainly metal NPs or other oxides/semiconductors). Supports are known to not only improve transferability and manageability of the nanomaterials concerning the practical catalytic process itself but in the ideal case also to improve the overall catalyst performance, for example, due to nanoparticle‐support interactions or an individual catalytic activity of the support which is further mediated by the NPs acting as co‐catalyst.^19^, ^296^, ^297^ To yield high mass‐based activities it is however generally mandatory to maintain a high dispersion and stabilization of the active species^297^, ^298^ as well as electrolyte accessibility and better conductivity in case of electrocatalysis.^299^ Especially the reducible oxides like TiO_2_, CeO_2_, V_2_O_5_, Nb_2_O_5_ are beneficial to also strongly interact with metal species, leading to Strong Metal Support Interaction (SMSI), resulting in special properties and altered or even improved catalytic performance.^300^ Indeed, the control of the interaction degree between the support and the active metal depends on pre‐ and post‐treatments to achieve the desired loading, size, electronic and bulk structure of the metal. Further, the search for suitable combinations of co‐catalyst and support is a key issue for many thermo‐ and electro‐catalytic processes.^301^

**Figure 15:**
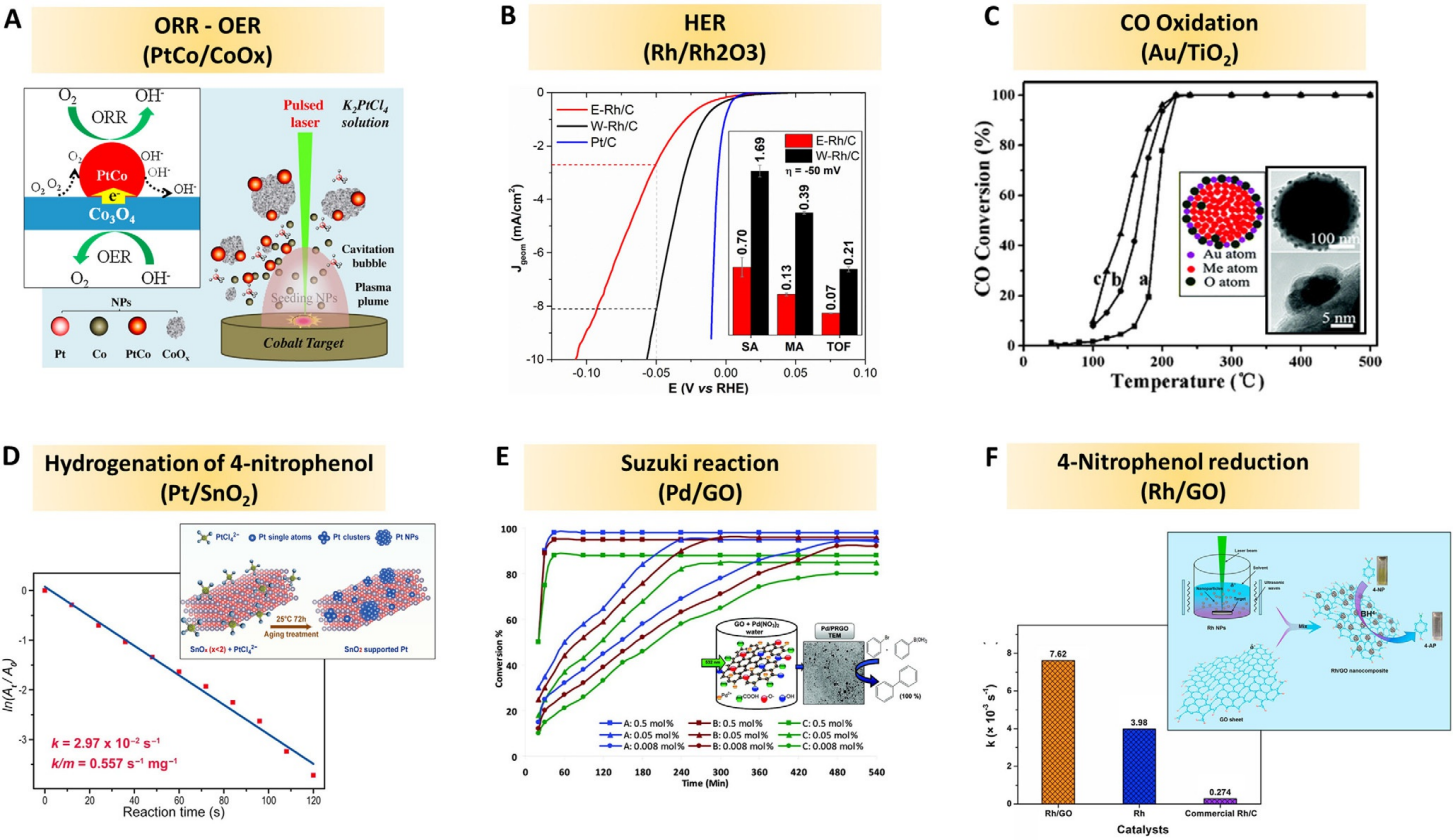
(A) PtCo/CoO_*x*_ catalysts prepared by laser ablation in tandem with galvanic replacement reaction for ORR and OER (reprinted with permission from ref. 191, Copyright 2016, Elsevier). (B) HER performances of Rh_2_O_3_‐coated metal Rh NPs in an acidic environment (E/Rh/C and W/Rh/C stand for NPs obtained, respectively in ethanol or water and supported on carbon electrodes; reprinted with permission from ref. 128, Copyright 2019, Royal Society of Chemistry). (C) CO conversion ratio as a function of temperature under the catalysis of Au/TiO_2_ heterostructures, where the curves a, b, and c correspond to one, two, and three cycles of catalysis, respectively. Inset shows a TEM image of Au NPs decorated iron oxide nanosphere and the sketch of the heterostructures obtained by this R‐LAL route (reprinted with permission from ref. 192, Copyright 2010, Royal Society of Chemistry). (D) The plot of ln(A_*t*_/A_0_) versus reaction time for catalytic hydrogenation of 4‐NP in presence of as‐prepared Pt/SnO2 catalysts (A_*t*_ is the absorbance at the characteristic peak of 4‐NP measured at time *t*). Inset shows the sketch for the synthesis of Pt/SnO_2_ NCs (adapted with permission from ref. 190, Copyright 2019, Elsevier). (E) Effect of concentration of the Pd/GO catalysts on the conversion of Suzuki cross‐coupling reaction of bromobenzene and phenylboronic acid. A, B, and C stand for samples prepared in pure water (*d*_nm_ Pd: 7.8), 50 % ethanol‐water (*d*_nm_ Pd: 10.8), and 50 % methanol‐water (*d*_nm_ Pd: 14.8), respectively (reprinted with permission from ref. 320, Copyright 2012, American Chemical Society). (F) Catalytic performance of Rh/GO on the reduction of 4‐Nitrophenol (reprinted with permission from ref. 321, Copyright 2019, Elsevier).

Oxygen reduction reactions (ORR), oxygen evolution reactions (OER) and water splitting processes, in general, require efficient and low‐cost catalysts as a part of the overall performance of fuel cells and batteries from the commercial point of view.^301^ Laser‐induced transformation of particles opens the way to increase the abundance of sites with tuned surface energy through the control of metastable and defective NPs. So far, pure iron oxides, as well as Co_3_O_4_ NPs produced by LAL, have been tested successfully when supported onto indium tin oxide (ITO) electrodes^302^ and unsupported.^303^ Besides, Hu et al.^191^ measured a combined ORR and OER overpotential of 756 mV versus RHE for PtCo/CoO_*x*_ catalysts prepared by laser ablation in tandem with galvanic replacement reaction (Figure 15 A), which is one of the highest values reported using carbon black as the supporting material. Very good catalytic performances in ORR were also demonstrated with perovskite LaMnO_3+δ_ 304 and Co_3_O_4_ 305 NPs synthesized by LAL.

In this context, oxygen vacancies introduced in CoOOH by LAL and LFL can strongly improve oxygen evolution reaction intermediates, showing a low overpotential of 330 mV at a current density of 10 mA cm^−2^, a small Tafel slope of 63.2 mV dec^−1^, and high catalytic durability, which all exceed those of commercial RuO_2_.^254^, ^306^ Similar defect formation scenarios and improved catalytic activity have also been observed apart from size reduction during LFL using ps‐532 nm‐laser pulses of CoO and Co_3_O_4_ 234 as well as CoFe_2_O_4_.^224^ Although the detailed defect formation mechanisms are not yet fully understood, these examples represent a promising basis for popularizing the use of laser technology in the design of defective oxide nanomaterials, for example, for application as efficient catalysts,^37^, ^224^, ^234^, ^254^, ^306^, ^307^ or plasmonic materials.^253^

More complex oxides and hydroxide nanostructures catalysts have been tested so far for similar applications. For instance, layered double hydroxides have shown to be active for water oxidation.^282^, ^308^ These compounds are based on mineral structures and can be easily found in nature.^282^ It is possible to obtain [NiFe]‐layered double hydroxides (LDH) with various intercalated anions such as Ti^4+^ or La^3+^ by using LAL, featuring very low overpotentials in the electrocatalytic water oxidation, as demonstrated by Hunter et al.^309^, ^310^ Another example is the synthesis of a noble‐metal free photocatalyst, namely CdS‐NiFe layered double hydroxide nanocomposite by using LAL.^311^ This nanocomposite exhibited a morphology of 2D‐NiFe LDH nanosheets on 1D‐CdS nanorods, providing an interfacial contact of heterostructures which allowed the efficient carrier transport and migration and a significant reactivity in the hydrogen evolution reaction. Such 2D oxide nanosheets are frequently used in catalysis. A recent example has been given by Thiago et al.^312^ in which graphene oxide (GO) and its reduced form act as a platform for covalently bonded sulfonic groups which enhance acid‐catalysed trans‐esterification reactions. GO also has excellent properties such as a high surface to mass ratio (1000 m^2^ g^−1^) and good solubility in water, then the chemical nature of functional groups and their concentration onto the graphene plane can be directly tuned in solution by laser irradiation.^313^ Among other 2D materials used in catalysis, it is noteworthy to mention bismuth sub‐carbonate (Bi_2_O_2_CO_3_). It is a member of Aurivillius related oxide family and has the interesting property to form 2D layers composed of orthogonally interleaved Bi_2_O_2_^(2+)^ layers with CO_3_^(2−)^ groups, held together by Van der Waals interactions along the *c*‐axis. In recent work, D'Angelo et al.^314^ have demonstrated the formation of Bi_2_O_2_CO_3_ nano‐sheets by UV laser irradiation of beta‐Bi_2_O_3_ in water, while in ethanol the process is accompanied by a partial reduction of the bismuth oxide, even containing zero‐valent Bi. Again, this highlights the importance of understanding the role of redox chemistry happening during laser‐based materials synthesis and processing. For example, the reactive oxygen species generated during LAL in water allows the achievement of oxidized noble metals like Rh_2_O_3_ coated metal Rh NPs, which exhibited high performances in the electrocatalytic hydrogen evolution (HER, Figure 15 B).^128^ Another interesting approach is the synthesis of catalytically active Au NPs/silica using a one‐step femtosecond‐reactive laser ablation in liquid (fs‐RLAL) technique.^315^ In this case, the use of fs laser pulses onto a silicon wafer immersed in an aqueous KAuCl_4_ solution allowed to obtain Au NPs with significantly smaller sizes than in previously reported RLAL studies, which were found to be highly active in the catalytic reduction of *para*‐nitrophenol. LAL synthesized NPs of various elements (Si, Ge, TiO_*x*_, SnO_*x*_, and MnO_*x*_) were used also as doping precursors for hydrothermal synthesis of hematite with improved photoelectrochemical performances.^316^, ^317^

In this frame, we like to recall the pioneering work by Lin et al.,^192^ one of the first attempts to use reactive laser synthesis in the liquid phase to obtain nanomaterials for catalytic application. In just one step, laser irradiation in liquid solutions can generate hybrid catalyst/support NPs in a metal‐core/oxide‐shell fashion like that reported in Figure 15 C. Such an „in situ“ reactive loading permits the formation of Ag/TiO_2_, Au/FeO_*x*,_ and Pt/FeO_*x*_ particles which have been successfully tested for carbon monoxide oxidation reactions (Figure 15 C). A crucial parameter, apart from the particle size, is the mass‐load of NPs on their support. For example, in case of the selective oxidization of ethanol using Au/TiO_2_ catalysts, an optimum of the absolute yield of acetic acid with the AuNP load has been found by Dong et al. which was hypothesized to be correlated with the coverage of all surface defects on TiO_2_ (e.g., oxygen vacancies) with laser‐generated AuNP.^318^ A similar conclusion was drawn by Jovic et al. in case of Au/TiO_2_ used for photocatalytic H_2_ evolution who correlated the optimum of catalytic activity and AuNP loading with the coverage of all „hot spots“ with NPs.^319^

In other examples, Pt loaded SnO_*x*_ has been proposed for the hydrogenation of 4‐nitrophenol (4‐NP).^190^ LAL of a metallic Sn target in a PtCl_4_^−^ solution generates a highly reactive environment with long‐living radicals, even after the ablation, which favours the formation of Pt clusters and the reactive loading of the catalyst onto the oxide support (Figure 15 D).

UV laser irradiation has been also used to build PdO/Pd/CNTs heterojunctions,^322^ with a controllable reduction degree and steerable PdO‐Pd interface. The nanostructures have been tested for the electrocatalytic N_2_ reduction reaction, where Pd absorbs N_2_ to form chemisorbed Pd‐N_2_ and PdO transmits protons to form α‐PdH, thus breaking N≡N triple bonds. The net result of the PdO/Pd interface enhanced the catalytic performance, coupling also low cost and an eco‐friendly way for ammonia synthesis under ambient conditions. Moussa et al. proposed a family of highly active Pd nanoparticle catalysts supported on partially reduced graphene oxide nanosheets for carbon‐carbon cross‐coupling reactions.^320^ There, pulsed laser irradiation of aqueous solutions of graphene oxide and palladium ions provides an excellent environment for anchoring the Pd NPs, thus hindering the migration of the particles and increasing the catalyst‐support interaction. With these catalysts, Suzuki, Heck, and Sonogashira cross‐coupling reactions have been tested with excellent results (Figure 15 E), with a turn over number (TON) of 7,800 and a remarkable turnover frequency (TOF) of 230 000 h^−1^ at 120 °C under microwave heating and good recyclability for Suzuki coupling.^320^

The electrostatic interaction of the catalysts with the 2D nano‐beds can be improved by external physical stimuli such as ultrasounds as reported by Liu et al.^321^ in the case of rhodium NPs (average size of 1.8±0.4 nm) decorated on graphene oxide nanosheets. The catalytic performance of Rh/GO on the reduction of 4‐nitrophenol is reported in Figure 15 F. Rate constants (*k*) for laser‐generated Rh/GO composites are 27 times higher than commercial Rh/C catalyst. Catalytic GO composites have been fabricated and tested also with Co_3_O_4_ NPs,^323^ TiO_2_ 324 and other NPs. Laser reduced graphene oxide has been also tested for direct dye removal from water solution and antibacterial activity.^325^, ^326^, ^327^ For instance, Russo et al.^325^ demonstrated that laser‐treated GO shows an excellent methylene blue (MB) adsorption capacity in water. Controlling the laser irradiation time between 10 and 250 minutes permitted to obtain GO sheets with different amounts of oxygen functionalities and, therefore, different degrees of reduction, affecting the hydrophilicity and the removal of MB. Further, compared to commercial catalysts, Haxhiaj and co‐workers observed a significantly higher tolerance of Pt@rGO (reduced GO) catalysts towards CO poisoning when the Pt NP were supported in situ during laser ablation of Pt in graphene nanosheet dispersion. The observation was discussed in terms of an enhanced interaction between rGO and Pt NPs due to a reaction of nascent and defect‐rich Pt NP with rGO surface directly after ablation.^328^

In conclusion, the synthesis of composites by laser‐assisted approaches in liquids appears a promising strategy to obtain active and stable species for a series of catalytic and photocatalytic applications, where NPs with defective structure and special surface characteristics are required to provide highly active and selective catalytic sites. The tuning of these properties, by controlling the parameters of the employed laser techniques, will be the main aim of the future research in this field, to expand and to optimize the performance of the synthesized systems.

### Bio‐applications

**Figure 16:**
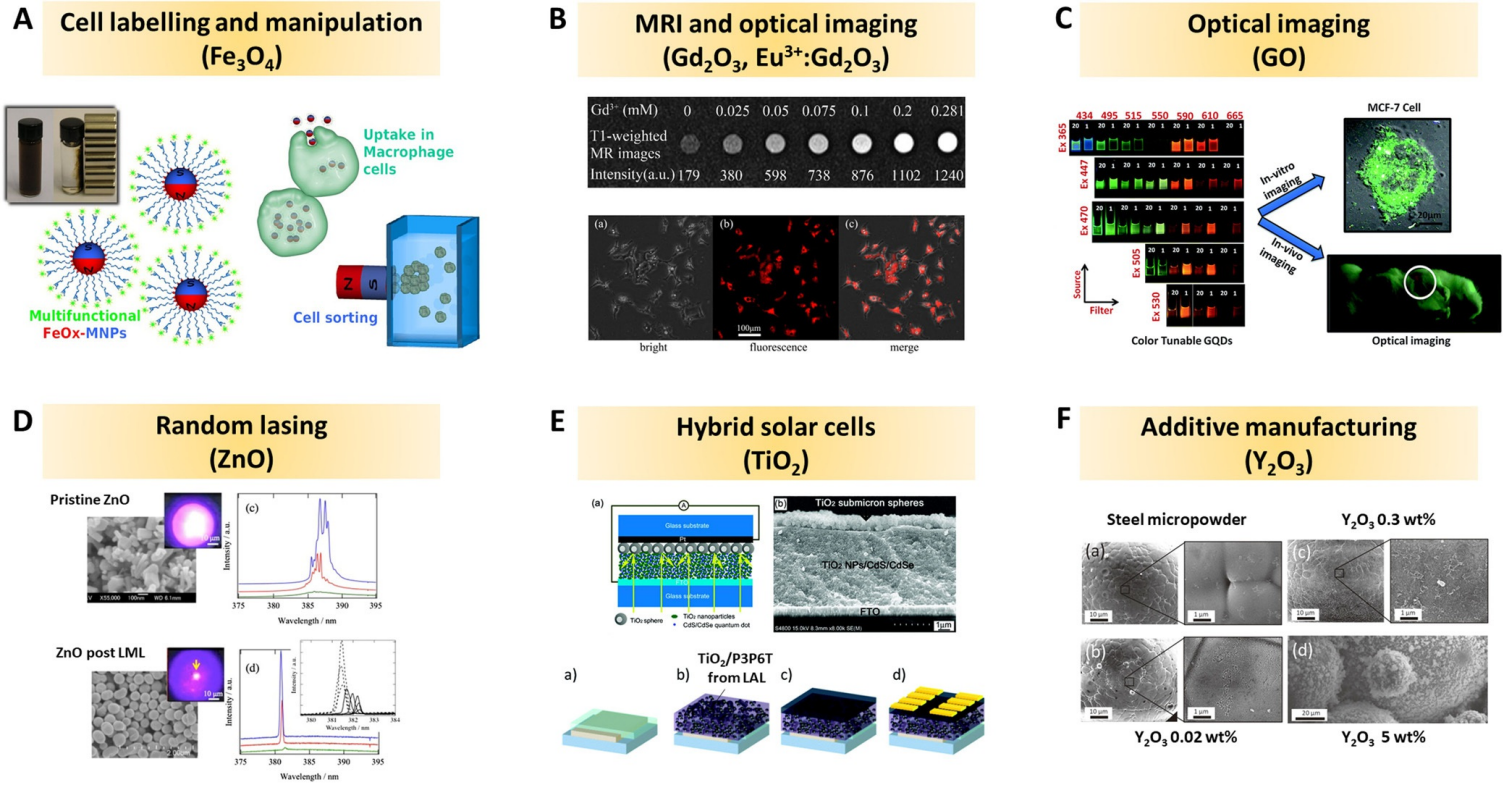
(A) Fluorescent iron oxide NPs are taken up by macrophages and allow magnetic cell sorting (reproduced with permission from ref. 118, Copyright 2011, Royal Society of Chemistry). (B) Gd oxide NPs provide efficient contrast for T_1_‐weighted MRI (upper panel, reprinted with permission from ref. 147, Copyright 2013, AIP Publishing). Eu^3+^ doped Gd oxide NPs associate fluorescence to MRI imaging properties, as shown in in vitro experiments (lower panel, republished with permission from ref. 331, Copyright 2018, IOP Publishing, Ltd. (C) GO quantum dots exhibit multispectral fluorescence and allowed in vitro and in vivo optical imaging (reprinted with permission from ref. 333, Copyright 2017, Royal Society of Chemistry). (D) ZnO microspheres with random lasing emission features, that are not observed in the raw ZnO powder before LML processing Adapted with permission under CC BY 4.0 from ref. 349, Copyright 2017, AIP Publishing). (E) Titania nanostructures were exploited as back reflectors of solar cells (upper panel, reprinted with permission from ref. 261, Copyright 2011, American Chemical Society) or for one‐step functionalization with water‐soluble P3P6T to easily achieve aqueous processed hybrid solar cells (lower panel, reprinted with permission from ref. 29, Copyright 2015, Royal Society of Chemistry). (F) Additive manufacturing: Y_2_O_3_ NPs homogeneously dispersed with iron‐chromium steel powders to obtain oxide dispersed strengthened alloy parts (reprinted with permission from ref. 361, Copyright 2018, The Japan Society of Applied Physics).

Oxide NPs have appealing properties for bio‐applications, thanks to the physical and chemical properties arising only at the nanoscale. The most common oxide nanostructure for biomedical purposes are iron oxides, and ultrapure non‐toxic laser‐generated NPs have been used for magnetophoretic sorting of cells and fluorescent cell labelling (Figure 16 A),^118^ or for doping of hydroxyapatite coatings of interest for biomedical devices.^329^ Phosphonate‐coated poly‐oxo‐clusters showed properties as T_1_ contrast agents for magnetic resonance imaging (MRI).^160^ Gd^147^ and Dy^330^ oxides have been also extensively investigated as T_1_ or T_2_ MRI contrast agents with promising performances (Figure 16 B). Doping of laser‐generated Gd_2_O_3_ oxide NPs with Eu^3+^ conferred photoluminescence properties in addition to the MRI contrast ability.^331^ Laser‐synthesized Si nanoparticles with variable level of surface oxidation showed appealing properties for biomedical applications thanks to their high purity.^332^

Graphene oxide quantum dots exhibited photoluminescence performances useful for bio‐imaging (Figure 16 C),^148^, ^333^ as demonstrated with long‐term tracking of cells in in vivo experiments.^333^ Besides, modification of graphene oxide by laser irradiation has been shown to enhance its antibacterial activity.^327^ NiO nanostructures have been also studied for antibacterial purposes.^334^

Of remarkable interest, a recent study showed the suitability of laser synthesis for implementation in a circular economy, by using waste battery cell powders to generate MnO_2_ NPs with excellent antibacterial properties.^335^

### Optics and photonics

In addition to photocatalysis and biophotonics, the optical properties of oxide nanostructures accessed by the laser‐assisted synthesis in liquid have stimulated the investigation and the application in different fields related to optics and photonics. For instance, photoluminescence properties of core–shell Si‐SiO_2_ NPs produced by pulsed laser ablation in aqueous solution were studied in detail, evidencing the presence of non‐radiative defects located in the suboxide interlayer between Si and SiO_2_.^336^ Core‐shell NPs made of LiNbO_3_ coated with a carbon shell exhibited blue‐green luminescence similar to carbon dots.^211^ Luminescence denoting quantum size range properties was reported in ultrafine ZnO nanocrystals obtained by LFL.^337^, ^338^ Strong second harmonic generation signals were measured in ZnO NPs obtained by the simple LAL procedure.^339^ Intense multi‐peak photoemission in the blue‐range was observed in Zn(OH)_*x*_‐DS layered composites obtained by LAL of Zn in aqueous SDS solutions. This behaviour is originated by the inorganic‐organic lamellar structure, as demonstrated also by scanning near‐field optical microscopy (SNOM) on a single Zn(OH)_*x*_‐DS nanosheet.^340^

Rare‐earth doped oxides have been synthesized for their emission properties,^341^, ^342^ including co‐doped Gd_2_O_3_ phosphors for upconversion, namely Gd_2_O_3_:Er,Yb^343^ and Gd_2_O_3_:Yb,Tm.^344^ Further, laser fragmentation has efficiently been employed to synthesize YVO_4_:Eu^3+^ which is another typical oxide relevant for upconversion.^345^

Nanocomposites of iron oxide and silver, obtained by S‐LAL, showed simultaneous properties of magnetism for magnetic attraction and plasmon resonances for spectroscopic detection of analytes by surface‐enhanced Raman scattering.^346^ Yttrium iron garnet NPs which are relevant for opto‐magnetic applications (e.g., magnetic circular dichroism) were gained with unexpectedly high magnetic coercivity behaviour by LAL.^101^ IrO_2_ nanocrystals, obtained by LAL in ethanol and subsequently supported on graphene oxide, exhibited peroxidase‐like behaviour useful for the colorimetric determination of ascorbic acid.^347^

Size‐specific optical applications for oxide particles fabricated by LML have been examined, including random laser using ZnO (Figure 16 D)^348^, ^349^ and back reflectors of solar cells using TiO_2_ (Figure 16 E),^261^ thanks to their appreciable sub‐micrometre size range and monodispersity. These applications require the strong scattering properties of dense sub‐micrometre particles having high refractive indices. Al_2_O_3_ NPs, generated by LAL in ethanol, were recently tested as a passivating and anti‐reflection coating for silicon photodiodes.^350^

Generation of TiO_2_ NPs by LAL was also exploited to promote their functionalization with water‐soluble poly[3‐(potassium‐6‐hexanoate)thiophene‐2,5‐diyl] (P3P6T), to easily achieve aqueous processed hybrid solar cells (Figure 16 E).^29^ LASiS of MoO_3_ NPs in water without undesired chemicals allowed easy spray coating deposition for the realization of solar cells.^140^ Piezo‐magneto‐plasmonic properties were reported for laser‐generated multi‐component PbZrTiO_3_/Au/Co nanostructures.^351^ While these are only a several examples of the application of LAL in optics and photonics the high potential of LAL in this field is evident.

### Other applications

Oxide nanostructures are ubiquitous in science and technology, and a series of specific applications have been proposed in the literature for laser‐generated oxide NPs, taking advantage of their purity, low production costs and biocompatible synthetic procedure. In the following, interesting examples are mentioned that go beyond catalysis, bio‐applications, and photonics.

Hydroponic experiments evidenced how CuO NPs with appropriate concentration enhance the growth of rice seedlings.^352^ Sub‐micrometre spheres of yttria‐doped ZrO_2_ prepared by LML showed anti‐wear properties in lubricants, where acted as submicron „ball bearings“ even after 3,000,000 wear cycles.^276^ Hence, such yttria‐stabilized zirconia (YSZ, widely used in dental implants because of self‐healing properties) NPs are useful as hard ceramic additives in lubricants oils,^271^, ^276^ and exceptional high particle strength for LML‐generated hard ceramic particles is confirmed.^353^

H_2_ gas sensing applications have been demonstrated for gold‐decorated graphene‐oxide/ZnO heterostructures.^354^ Selective ethanol gas sensing performances were reported for CuO nanocrystals,^355^ ZnO NPs,^356^ and Au/ZnO nanospheres.^357^

ZnO nanocrystals exhibited a selective absorbing capacity for toxic or carcinogenic volatile aromatic compounds such as aniline,^260^ and laser synthesis in liquid was used also for the preparation of oxide NPs (barium hexaferrite,^358^ tin oxide,^359^ or alumina^360^) for surfactant‐free nanofluids.

The rising of additive manufacturing has benefited from depositing laser‐generated Y_2_O_3_ NPs on iron‐chromium powders to obtain oxide dispersed strengthened alloy parts by laser powder bed fusion (often called selective laser melting SLM) (Figure 16 F).^361^

## Summary and Outlook

A wide variety of oxide nanostructures exist, each one with important properties for scientific and technological applications. Therefore, it is of utmost importance to realize the synthesis of oxide NPs by environmentally friendly, energy‐saving, simple and effective routes. In this review, we showed that laser‐assisted synthesis in liquid environment is a versatile approach running at room temperature and pressure, with highly encouraging results in terms of purity of products and absence of undesired chemical compounds or pollutant wastes. Laser‐assisted synthesis allows productivities up to the g/h‐scale of NPs, while the synthesis of low‐priced oxide materials and all those cases demanding > kg/h‐scales today are sometimes better achieved by other routes such as gas‐phase synthesis, at least if aggregation (that is inherent to gas phase synthesis) is not hindering application. Gram scale productivities are more difficult to reach compared to (noble) metal ablation as mass productivity also scales with the density of the material.^43^ Also, with only few exceptions, oxides generally show weaker absorbance in the infrared range, so shorter wavelengths and/or pulse durations are required for efficient LAL, LFL, LML, and by that more expensive laser systems are required for up‐scaled laser synthesis of oxides. Conversely, laser approaches were shown to be economically preferable over conventional synthesis protocols for high value‐added nanomaterials and are worthy of consideration for advanced fundamental studies or for benefits that go beyond the economic balance. For instance, the integration of laser‐assisted synthesis of oxides in a circular economy has been also envisaged in the literature.^234^, ^335^ Yet, with the laser‐based productivity of NPs having been scaled of more than 3‐orders of magnitude (from less than mg h^−1^ to g h^−1^) within the last decade, a further increase is foreseeable. In the future, this increase may be achieved by smarter beam handling strategies such as parallel processing, automation measures for target feeding (as target feeding on the multi g/h‐scale still presents a bottleneck) and control systems,^34^, ^39^, ^40^ as well as commercial high‐power, ns and ps pulsed laser systems steadily are getting cheaper year by year. Note that this upper‐end productivity limit values are not hindering application or Technology Readiness Level of the method in general. For example, bio‐application mass demand in diagnosis or therapy (e.g., with laser‐generated bioconjugates or SiO_2_) is met already with today's throughput level. But not only the upper‐end productivity value is a (commercialization) limitation of laser synthesis in liquid, compared with the conventional routes for the synthesis of oxides. On the one hand, laser synthesis has the advantage to provide aggregation‐free colloidal nanoparticles, different from gas‐phase synthesis or hydrothermal synthesis. On the other hand, the produced colloid often has a comparable low concentration (some 100 mg l^−1^ instead of several g l^−1^) to avoid beam attenuation effects. This naturally lower concentration limit of LAL and LFL (that may be higher for LML and LDL) requires more post‐processing efforts as the liquid has to be removed for the final products. Another limitation is the understanding to synthesize multi‐element oxide materials with better control in stoichiometry, like in hydrothermal synthesis of doped or multi‐element oxides. Laser synthesis community is quite active in the fundamental research level as outlined in the related chapters above, but in the literature the yield of a specific doped/multi‐element oxide (referred to by‐products with different stoichiometry) is often not described, same for the particle size dependency of the oxide nanoparticle composition. Here size quenching methods that do not rely on organic ligands (that would compromise one of the key advantages of laser synthesis) are required that reach a high yield of for doped and multi‐element oxides with monodisperse size.

Only two experimental set‐ups (laser ablation of solids and laser irradiation of colloids) allowed the access to a library of oxide NPs (spheres, rods, flowers) in one step, which also includes metal‐oxide core–shells or heterostructures. As reviewed in this paper, the large part of oxide nanostructures can be obtained all by LAL with near‐infrared (e.g., 1064 nm) ns laser pulses in pure liquids, that makes very easy to switch from one type of material (also non‐oxide) to another in a „plug‐and‐play“ procedure. On the other hand, the choice of the main parameters, such as the synthetic approach (LAL, LFL, LML, LDL), the composition (oxide, metal, alloys), and type (plate, powder, colloid) of the starting material, the liquid type, the solutes, the laser parameters (wavelength, pulse duration, fluence) can be used to control the size, composition, phase and sometimes morphology of the products. Aiming at fine‐tuning oxide properties and defect doping, the emerging field of laser defect engineering in liquid (LDL) is expected to provide new insights on structure–activity correlations in catalysis research.

Although the LAL, LFL, LML, and LDL procedures and effectiveness have been sensibly developed in the past few years, the fundamental processes are still under debate, which is leading to a close collaboration of experimentalists and theoreticians to reach the same level of understanding and control as already achieved by the chemical nanoparticle synthesis routes. The theoretical and experimental understanding of the whole synthetic process is still undergoing, especially for what concerns the challenging and elusive steps of high‐energy laser pulses‐matter interaction, plasma/liquid interactions, and chemical reaction of ablated matter with solution species, as well as the gradient of thermodynamic parameters from the centre of the laser spot to its boundaries.

Going deeper into our understanding is crucial to improve the control over the particle size distribution obtained by LAL, that is more difficult than with chemical routes unless when they are post‐processed with LFL or LML. For example, the LAL synthesis of quinary high extropy alloy at the gram scale has recently been shown with a dominant small particle size fraction, but still, large particles appear that compromise the mass yield and catalytic performance.^362^ The identification of synthesis conditions that avoid size polydispersity, non‐crystalline products and coexistence of different phases is important also to unify the multiple laser synthesis parameters among the variety of solvents, target materials, laser pulse wavelength, duration, fluence and repetition rate. To date, this variety was useful to show all the potentialities of laser‐assisted synthesis but also delayed the worldwide diffusion of the best production protocols. An effort in that direction has recently been made by publishing recipes of best practice in laser synthesis.^21^

The solution to these problems will likely lead laser‐generated oxide nanostructures to real products in the market, given the overall series of advantages inherently associated with these synthetic approaches.

## Conflict of interest

The authors declare no conflict of interest.

## Biographical Information

*Vincenzo Amendola is Associate Professor of Physical Chemistry at Padova University, where he obtained the PhD in materials science and engineering in 2008 and the Italian qualification as full professor in 2017, after research experience at M.I.T. and Cambridge University. With his Laser Assisted Synthesis and Plasmonics lab, he searches for unconventional and non‐equilibrium nanomaterials exploitable for experimental and theoretical investigations in plasmonics, sensing, nanomedicine and catalysis*.

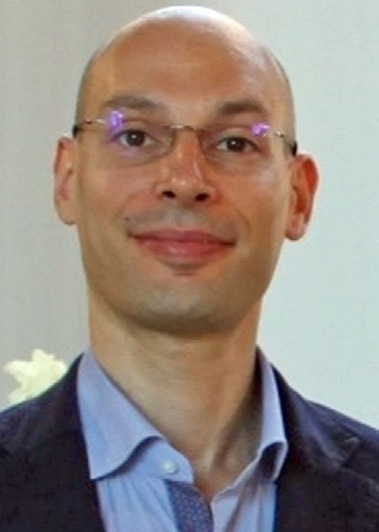

## Biographical Information

*David Amans is Associate Professor of physics at University Claude Bernard Lyon 1. He studied computing and materials science at Centrale Lyon from where he also received his Ph.D. in 2002. As a postdoc, he worked at University Libre de Bruxelles on quantum information and nonlinear fibre optics, and then at PHELMA school on optronics. He joined the Institute of Light and Matter in 2005 where he is developing laser ablation in liquids, addressing nanomaterials science and laser‐induced plasma*.

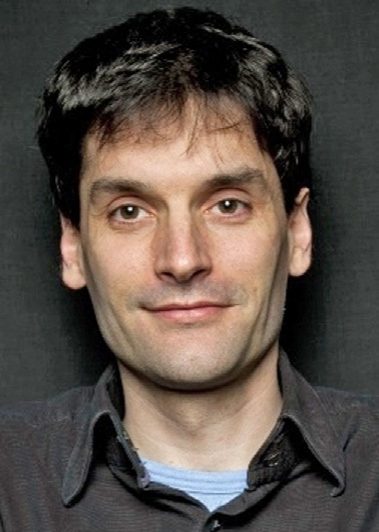

## Biographical Information

*Yoshie Ishikawa is a Senior Researcher at Nanomaterials Research Institute, National Institute of Advanced Industrial Science and Technology (AIST) since 2013. She obtained her Ph.D. from Kumamoto University in 2003 and was an Associate Professor in Kagawa University until 2013. Her scientific focus is on the fabrication and application of pulsed laser melting in liquid for metallic and inorganic sub‐micrometre particle synthesis*.

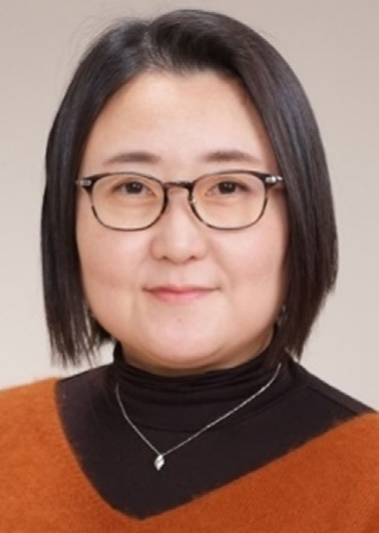

## Biographical Information

*Naoto Koshizaki currently is Guest Professor in the Division of Quantum Science and Engineering of the Graduate School of Engineering in Hokkaido University. He obtained his Ph.D. from University of Tokyo in 1997. Until 2013 he was a Senior Researcher in the National Institute of Advanced Industrial Science and Technology. Currently he works on physical fabrication methods for inorganic nanoparticles and nanocomposites*.

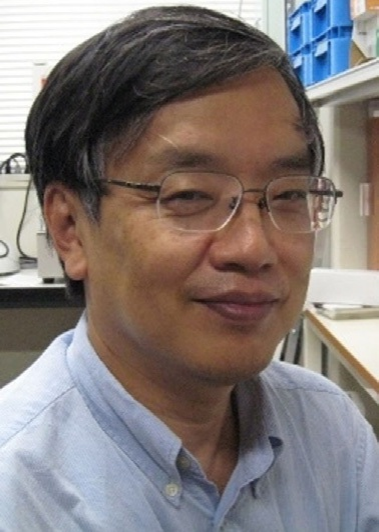

## Biographical Information

*Salvatore Scirè is an Associate Professor of Industrial Chemistry at the Department of Chemical Sciences of Catania University (Italy). His research activity is focused on heterogeneous catalysis, with special interest in oxide‐supported mono and bimetallic catalysts, and more recently to the application of photocatalysis to environmental protection and energy production. His activity is documented by about 100 papers in international journals and books, 1 patent, and over 110 contributions in scientific meetings*.

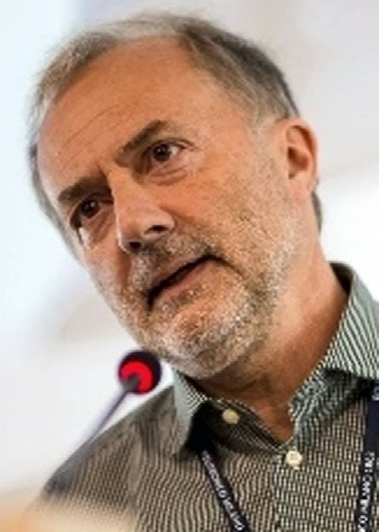

## Biographical Information

*Giuseppe Compagnini is Full Professor of Physical Chemistry at the University of Catania. His research group focuses on fundamental and applied aspects of nanocomposites, including laser ablation, micro‐ and nano‐joining, and vibrational spectroscopy. He is head of the Thin films and Nanostructures laboratory at the Department of Chemical Sciences and Head of the PhD School of Materials Science and Nanotechnology. He is author of about 160 papers on international peer reviewed journals (ISI) and 10 invited reviews, receiving more than 5000 citations*.

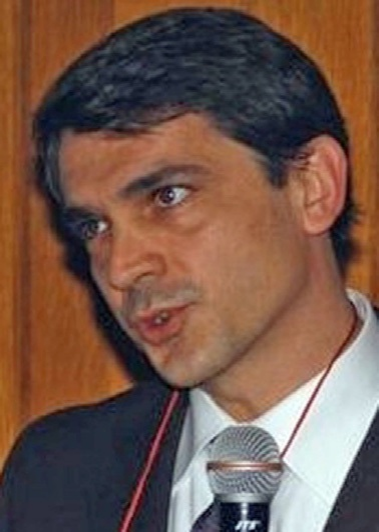

## Biographical Information

*Sven Reichenberger is acting leader of the catalysis research group at the Institute of Technical Chemistry of the University‐Duisburg Essen. He received his Ph.D. in 2017 at the University of Duisburg‐Essen and specialized in the field of laser‐based defect engineering during a post‐doctoral research. He currently focuses on his habilitation on surface processes occurring during laser‐based catalyst synthesis, with a focus on fuel cells, electrolyzers, and oxidation catalysts*.

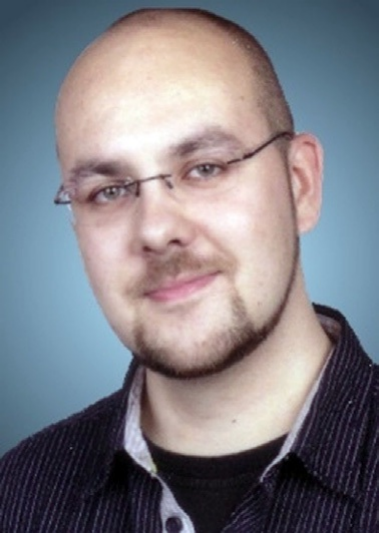

## Biographical Information

*Stephan Barcikowski is Full Professor and Chair of the Institute of Technical Chemistry I at the University of Duisburg‐Essen. In 2004 he received his Ph.D. in Mechanical Engineering, followed by his habilitation in Chemistry on laser‐generated nanomaterials in 2011. His research targets the nanoparticle formation mechanisms in laser ablation and fragmentation, as well as their upscaling aiming at their application in catalysis, biomedicine, and additive manufacturing*.